# Non-invasive assessment of stroke volume and cardiovascular parameters based on peripheral pressure waveform

**DOI:** 10.1371/journal.pcbi.1012013

**Published:** 2024-04-18

**Authors:** Kamil Wołos, Leszek Pstras, Malgorzata Debowska, Wojciech Dabrowski, Dorota Siwicka-Gieroba, Jan Poleszczuk

**Affiliations:** 1 Laboratory of Mathematical Modeling of Physiological Processes, Nalecz Institute of Biocybernetics and Biomedical Engineering, Polish Academy of Sciences, Warsaw, Poland; 2 Department of Anesthesiology and Intensive Therapy, Medical University of Lublin, Lublin, Poland; University of California Riverside, UNITED STATES

## Abstract

Cardiovascular diseases are the leading cause of death globally, making the development of non-invasive and simple-to-use tools that bring insights into the state of the cardiovascular system of utmost importance. We investigated the possibility of using peripheral pulse wave recordings to estimate stroke volume (SV) and subject-specific parameters describing the selected properties of the cardiovascular system. Peripheral pressure waveforms were recorded in the radial artery using applanation tonometry (SphygmoCor) in 35 hemodialysis (HD) patients and 14 healthy subjects. The pressure waveforms were then used to estimate subject-specific parameters of a mathematical model of pulse wave propagation coupled with the elastance-based model of the left ventricle. Bioimpedance cardiography measurements (PhysioFlow) were performed to validate the model-estimated SV. Mean absolute percentage error between the simulated and measured pressure waveforms was 4.0% and 2.8% for the HD and control group, respectively. We obtained a moderate correlation between the model-estimated and bioimpedance-based SV (r = 0.57, p<0.05, and r = 0.58, p<0.001, for the control group and HD patients, respectively). We also observed a correlation between the estimated end-systolic elastance of the left ventricle and the peripheral systolic pressure in both HD patients (r = 0.84, p<0.001) and the control group (r = 0.70, p<0.01). These preliminary results suggest that, after additional validation and possibly further refinement to increase accuracy, the proposed methodology could support non-invasive assessment of stroke volume and selected heart function parameters and vascular properties. Importantly, the proposed method could be potentially implemented in the existing devices measuring peripheral pressure waveforms.

## Author summary

With a growing number of people suffering from cardiovascular diseases (CVD), it is extremely important to develop non-invasive methods that could quickly and comprehensively assess the condition of the cardiovascular system. Hemodialysis (HD) patients are particularly prone to CVD, but studies aimed at developing new techniques for non-invasive assessment of cardiovascular parameters are typically based on healthy subjects. We set out to investigate the possibility of using peripheral pressure waveforms to estimate stroke volume (SV) and selected parameters of the cardiovascular system in both healthy subjects and HD patients. To that aim, we developed a mathematical model of pulse wave propagation coupled with an elastance-based model of the left-ventricular function. The validity of the model has been assessed using bioimpedance-based SV estimates from both healthy subjects and HD patients. We believe that the proposed model-based procedure, subject to further validation and improvements, could serve as an auxiliary tool for evaluating SV. In addition, it enables estimation of selected cardiovascular parameters, some of which, as we have shown, appear to be related to known pulse wave-derived parameters.

## Introduction

Chronic kidney disease (CKD) is a long-term decrease in kidney function characterized by its progressive nature, lack of a definitive cure, and high morbidity and mortality. It is relatively common in the general adult population, particularly among individuals affected by diabetes and hypertension [1]. Prevalence of CKD is estimated at 13.4% globally and its incidence is expected to rise in the future [2,3]. About 50% of patients with CKD stage 4 or 5 have a cardiovascular disease (CVD), with approximately 40% to 50% of all deaths in this group being attributed to CVD [2]. CVD risk factors in patients with CKD include, among others, vascular calcification, inflammation, hypertension, or diabetes [2]. Furthermore, nearly half of patients with heart failure suffer from CKD [4] and a long-term decrease in cardiac output or stroke volume may indicate progression of heart failure, which can decrease kidney perfusion and lead to kidney failure [5,6]. Non-invasive methods of assessing the patient’s cardiovascular health can be an important aid in understanding the CKD-related mechanisms behind CVD, predicting hemodynamic response to various treatments, or creating personalized treatment plans. Moreover, non-invasive assessment of stroke volume in end-stage CKD patients receiving dialysis treatment could potentially help in assessing the fluid overload in such patients and prescribing an adequate and safe dialysis treatment. It could also be used for categorizing dialysis patients based on their intradialytic hemodynamic changes [7]. For these reasons, it is critically important to develop easy-to-use and non-invasive methods of assessing the patient’s cardiovascular condition.

In recent years, personalized cardiovascular mathematical models of various complexity have begun to play an increasingly important role in the assessment of the state of the patient’s cardiovascular system [8–13]. Coupled 0-1D models are a good compromise between the simplicity of lumped models and the complexity of multidimensional models in the case when one is interested in describing the whole-body circulation. In this type of reduced-order modeling, the pulse and flow wave propagation are described using a one-dimensional bifurcation tree reflecting the largest arteries in the body, on which the flow equations are imposed. In such models, the lumped parts typically explain the behavior of the peripheral arteries and the work of the heart. The size of the modeled system, as well as the parameters describing the heart and the vasculature (i.e. peripheral compliance, resistance, etc.) are subject-specific and should be estimated for a given patient. To personalize a model, one needs to collect (preferably non-invasively) patient data corresponding to the model outputs and then to adjust the model (i.e. optimize the model parameters), so that its outputs match gathered data as closely as possible. A proper optimization method, combined with appropriately assigned initial values of the parameters to be optimized (e.g. based on patient size, age, gender, etc.), should minimize the difference between the model outputs and patient data, providing the final estimates of patient-specific values of those parameters, thus making the model personalized. Depending on the application and the details of the model, various approaches to model personalization may be used.

For instance, in the study by Zang et al [14] a 0-1D model describing 55 main arteries (defined previously in the works of Stergiopulos et al. [15] and Olufsen et al. [16]) was personalized by scaling the artery lengths and diameters according to subject height and a cluster-dependent scaling factor, followed by minimizing the error between the measured and simulated peripheral pressure waveforms using the gradient-based optimization algorithm. In the study by Bikia et al. [11] a model based on a more detailed bifurcation tree with 103 arteries (defined previously by Reymond et al. [17]), was personalized by: 1) adjustments of the arterial tree based on age, gender, height, and body surface area; 2) using a gradient-based optimization procedure to minimize the error between the measured and computed cardiovascular variables such as systolic and diastolic blood pressure, and carotid-femoral pulse wave velocity. Carson et al. [18] used a two-tier optimization procedure involving adjustments of parameters such as peripheral resistances, compliances, blood volume, and arterial cross-sectional areas to fit the model to the measured systolic and diastolic blood pressure, pulse wave velocity, as well as peak systolic and end-diastolic blood flow velocity.

Once the model is personalized, the resulting set of subject-specific parameters can provide deeper insight into the patient’s condition without the use of invasive measurements [9–11,19]. However, to our knowledge, not many of such studies have been conducted in patients with chronic diseases such as CKD, who, as already mentioned, are particularly prone to CVD.

In our previous papers, we have investigated whether a 0-1D model could reproduce applanation tonometry recordings of pressure waves from the radial artery in healthy subjects and hemodialysis (HD) patients [10,19]. We also performed a detailed study on whether the cardiovascular risk factors for HD patients, such as arterial stiffness, could be derived from the measured pressure waveforms [19]. In the present paper, we extend our previous study by investigating if the measured pressure waveform can be used for a detailed heart function assessment, including stroke volume (SV) estimation. We use a similar 0-1D model as before i.e. the model based on the aforementioned commonly-used 55-element arterial tree and a 3-element Windkessel model at the terminal ends of the arteries. However, we decided to use a different boundary condition describing the inflow of blood to the arterial tree, which in the present study is based on the elastance function of the heart. To validate the model-based estimates of SV, we used bioimpedance cardiography measurements (PhysioFlow, Manatec Biomedical, France) performed simultaneously with peripheral pulse waves recordings.

## Results

### Reproduction of the measured pressure waveforms

**Fig 1:**
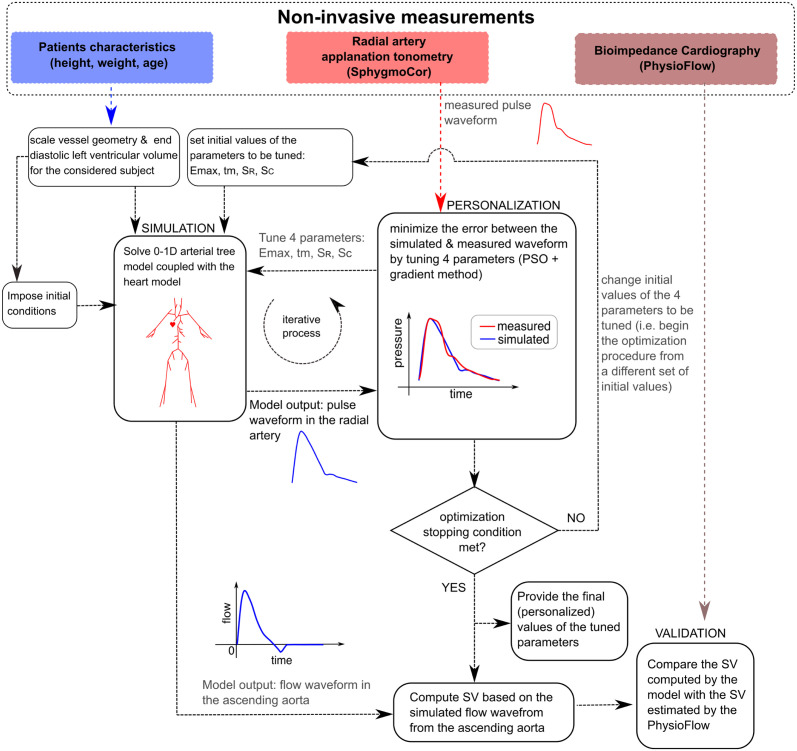
Simplified workflow of the study. For each measured pressure waveform, model personalization (calibration) was performed. An iterative optimization procedure was employed to tune the values of parameters describing the function of the heart (i.e. *E*_*max*_–maximal value of the elastance function, and *t*_*m*_–time to the onset of constant elastance) as well as terminal compliances and resistances (*S*_*c*_ and *S*_*R*_, respectively) that would minimize the error between the measured and simulated pressure waveform in the radial artery. After model personalization, the model-simulated blood flow waveform in the ascending aorta was used to estimate stroke volume (SV). Finally, model-estimated and reference SV values have been compared.

**Fig 2:**
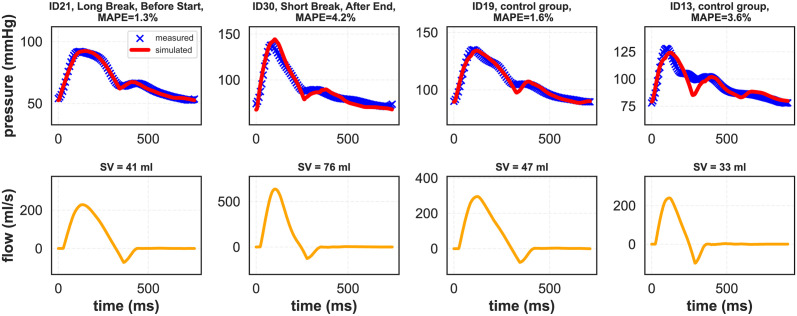
Exemplary model simulations of the pressure waveform in the radial artery (upper panels) and the corresponding blood flow waveform in the ascending aorta (lower panels) in four subjects: Two HD patients and two subjects from the control group.

**Fig 3:**
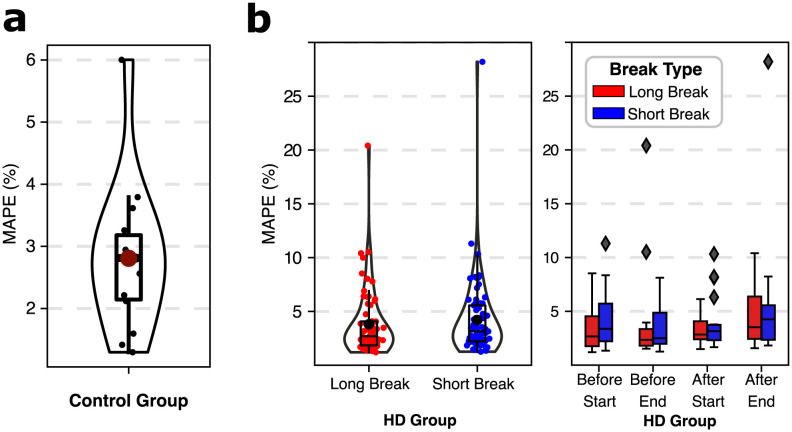
The quality of model fits: Mean absolute percentage error (MAPE) between the measured and model-simulated pressure waveforms in the radial artery for (a) control group and (b) HD patients. For HD patients, the results are divided based on either the length of the interdialytic break before the HD session (a long, 3-day break vs short, 2-day break) or the time of the measurement (before/after the start of the HD session and before/after the end of the session).

After subject-specific personalization (calibration) of the model (see Fig 1 for a graphical representation of the calibration process), our pulse wave propagation model was able to reproduce the pressure waveforms recorded in the radial arteries with satisfactory accuracy in most cases. Exemplary model simulations after data fitting are presented in Fig 2, whereas all cases are presented in the S1 File. Additionally, S1 Fig shows exemplary model outputs at different locations in the arterial tree (i.e. flow and pressure waveforms). For the control group, the average mean absolute percentage error (MAPE) between the measured and fitted pressure waveforms (for all available data points) was 2.8%, whereas for HD patients it was 4% (see Fig 3 for more details). The average MAPE for the measurements performed during HD after a long interdialytic break (i.e. 3 days) was slightly lower compared with that obtained for the measurements performed after a short, 2-day break (3.8% vs. 4.2%; difference not significant), see Fig 3b. The worst (highest) MAPE values were obtained for the measurements conducted after the end of HD (average 4.9% for all HD sessions, 4.4% for the sessions after the long interdialytic break, and 5.5% for the sessions after the short interdialytic break; difference not significant).

### Stroke volume estimation

**Fig 4:**
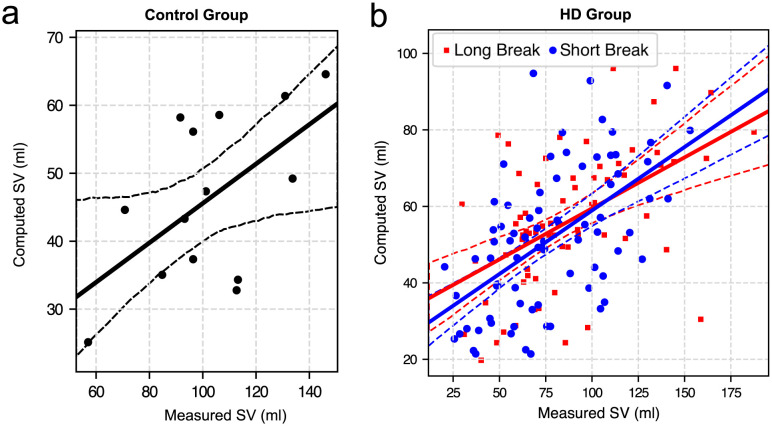
Comparison between the model-estimated (computed) stroke volume (SV) and bioimpedance-based (measured) SV values for (a) control group and (b) HD patients (data shown separately for the HD sessions after a long and short interdialytic break). Solid and dashed lines represent linear regression, and 95% confidence intervals, respectively.

For the control group, the Pearson correlation coefficient between SVs estimated by the bioimpedance cardiograph and those estimated using our model was r = 0.57 (p = 0.032), see the scatter plot in Fig 4a. For HD patients, the correlation coefficient for all measurements was r = 0.58 (p < 0.001), with r = 0.56 and r = 0.59 for the measurements performed after the short and long interdialytic break, respectively (p < 0.001 in both cases; see Fig 4b for the scatter plots). The correlation coefficients were similar regardless of the time of measurement during the HD session, with the only exception for the measurements performed before the end of HD session after a long interdialytic break, where the correlation was higher (r = 0.75, p < 0.001), although only after excluding one clear outlier, see S2 Fig.

**Fig 5:**
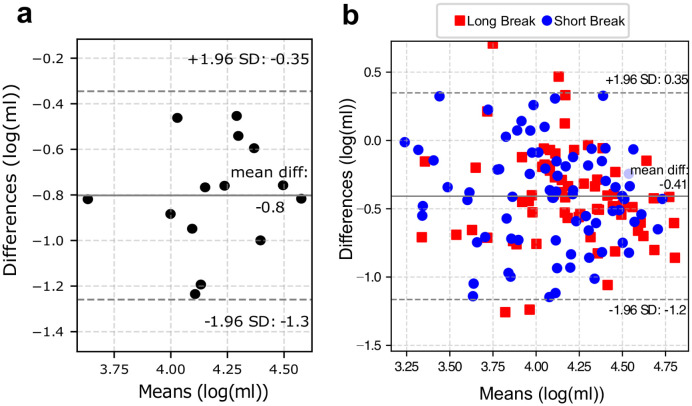
Bland-Altman plots comparing the stroke volume estimated by the model versus estimated using bioimpedance cardiography (PhysioFlow) for (a) control group and (b) hemodialysis patients. Dashed horizontal lines represent the 95% limits of agreement, straight horizontal line represents the mean difference. Before plotting, the data have been logarithmically transformed. SD denotes standard deviation.

Fig 5 presents the Bland-Altman plots comparing the model-estimated and bioimpedance-based SV values in both control and HD groups. Since the differences in SV from the two methods were not normally distributed in the HD group (Shapiro-Wilk test statistic: 0.98, p < 0.05), a logarithmic transformation of the data was performed [20] (Shapiro-Wilk test statistic: 0.991, p = 0.52).

### Estimation of cardiovascular parameters

**Fig 6:**
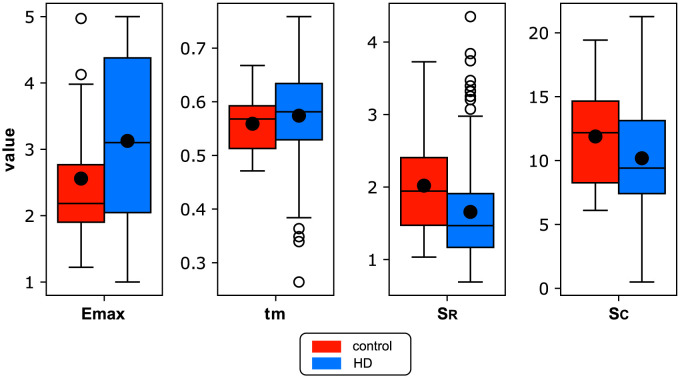
Box-plots for the estimated cardiovascular parameters in the hemodialysis (HD) and control groups. The description of parameters and their units are provided in Table 1.

Table 1 presents the average values of the estimated subject-specific parameters and their standard deviations. The first two parameters are related to the elastance heart model of the left ventricle, while the last two describe the properties of the terminal vascular beds, (see also Fig 6 to compare).

**Table 1:** Summary of the estimated patient-specific values of the model parameters for hemodialysis (HD) patients and control group. The data are shown as means and standard deviations (SD).

Parameter		Unit	HD patients		Control group	
			Mean	SD	Mean	SD
*E*_*max*_	Maximal systolic value of elastance function	mmHg/ml	3.13	1.27	2.56	1.06
*t*_*m*_	Time to the onset of constant elastance	s	0.57	0.09	0.56	0.05
*S*_*R*_	Scaling factor of terminal resistances	-	1.66	0.71	2.02	0.75
*S*_*c*_	Scaling factor of terminal compliances	-	10.18	4.34	11.89	4.39

Table 2 shows the comparison between the mean values of the cardiovascular parameters estimated in HD patients for the measurements performed after the long vs short interdialytic break. This comparison is shown in two versions: first, for the available data from all four measurement moments during HD, and second, for the measurements taken only before the start of HD (to exclude the impact of the dialysis itself). In both cases, when paired measurements were taken into account, no statistically significant differences were observed with regard to the length of the interdialytic break.

**Table 2:** Summary of the estimated values of the cardiovascular parameters in hemodialysis (HD) patients depending on the length of the interdialytic break before the studied HD session (a long, 3-day break vs a short, 2-day break). The data are presented as means (± standard deviation).

Parameter	Unit	All measurement moments^1^		Before Start^2^	
		long break	short break	long break	short break
*E*_*max*_	mmHg/ml	2.99±1.13	2.89±1.24	3.43±1.06	3.44±1.24
*t*_*m*_	s	0.58±0.09	0.58±0.09	0.58±0.06	0.58±0.08
*S*_*R*_	-	1.57±0.65	1.77±0.75	1.51±0.50	1.71±0.91
*S*_*c*_	-	10.93±4.70	10.49±3.99	11.82±4.17	11.41±4.66

^1)^ the results combine data from all moments of measurements, i.e. before and after the start of the HD session and before and after the end of the session, but limited to cases for which the data for the given patient and the given moment of measurement was available for both HD sessions (i.e. paired data only)

^2)^ the results refer to measurements performed before the start of the HD session but limited to cases for which the data for the given patient was available for both HD sessions (i.e. paired data only)

### Correlations with PWA-derived indices

**Fig 7:**
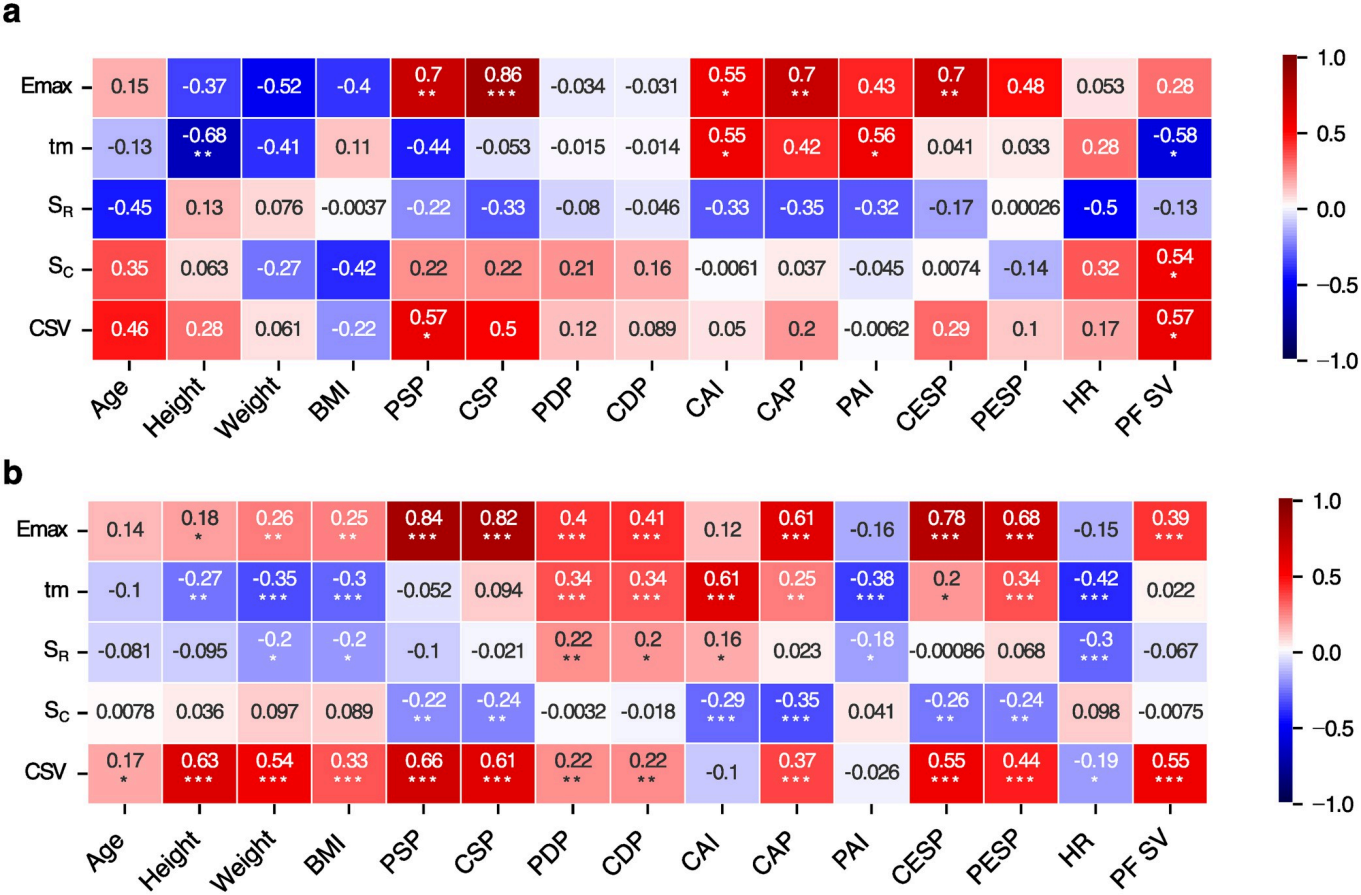
Correlation matrix between the parameters estimated by the model (Y axis) and cardiovascular parameters or indices derived by SphygmoCor (X axis) for (a) control group of healthy subjects and (b) hemodialysis patients, * p < 0.05, ** p < 0.01, *** p < 0.001. CSV–computed stroke volume, BMI–body mass index, PSP–peripheral systolic pressure, CSP–central systolic pressure, PDP–peripheral diastolic pressure, CDP–central diastolic pressure, CAI–central augmentation index, CAP–central augmentation pressure, PAI–peripheral augmentation index, CESP–central end-systolic pressure, PESP–peripheral end-systolic pressure, PF SV–stroke volume estimated by PhysioFlow.

The SphygmoCor device, which was used for peripheral pressure waveform recordings, performs also the so-called Pulse Wave Analysis (PWA), which is a non-invasive method of assessing the state of the cardiovascular system. The PWA method relies on a generalized transfer function that allows reconstruction of the central pressure waveform from that recorded in the radial artery. Thus, the device also returns parameters characterizing the peripheral and central pressure waveforms, such as the augmentation index. To determine whether the model-estimated SV and the cardiovascular parameters tuned in our model fitting procedure are somehow related to the indices derived by SphygmoCor and the general patient characteristics (age, height, weight), we calculated the Pearson correlation coefficients between those values and presented them in the form of a heatmap, see Fig 7.

## Discussion

We presented a model-based methodology for estimating SV based on the pressure waveform recorded in the radial artery. We observed a moderate correlation between the SV estimated by our model and that obtained from bioimpedance cardiography in both healthy subjects and HD patients (Pearson correlation coefficient of around 0.6). However, our model-based estimates of SV were generally lower than those obtained from bioimpedance cardiography, which may be either due to the limitations of our model or due to imperfectly recorded peripheral pressure waveforms to which our model was fitted, or due to inaccuracy of the reference (bioimpedance-based) SV values (or a combination of the above). For instance, in some cases PhysioFlow provided SV values that could be considered unrealistic (e.g., values over 150 ml, see Fig 4b and S2 Fig). The model, on the other hand, operates on physiological parameters, such as the maximum left-ventricular volume (properly scaled for the given patient), and thus is unable to provide such unrealistically high values of SV. Nevertheless, ignoring the cases with questionable reference data (SV over 150 ml) did not significantly affect the correlation coefficients.

PhysioFlow employs neck and chest electrodes to measure changes of the thorax impedance induced by pulsatile blood flow generated by the heart, thus enabling the estimation of SV. Compared to the well-established echocardiography, bioimpedance cardiography is less demanding when cardiovascular parameters need to be evaluated multiple times during an HD session [21]. Echocardiography, which uses ultrasound waves to obtain images of the heart structure and function must be operated by a qualified clinician. Given that in our study for each HD session we needed four reference measurements (estimations) of SV performed over the span of approximately 4 hours, we decided, therefore, to use PhysioFlow, which did not require qualified personnel and hence was much easier and cheaper (the electrodes were attached to the patient before the HD session and were kept on for the whole session). A disadvantage of this approach is the aforementioned possible inaccuracy of bioimpedance cardiography in estimating SV–even though some studies report good accuracy of this method, including in HD patients, both at the beginning and at the end of HD [21,22], there are also studies that questioned it in certain groups of patients, such as anemic or pediatric patients [23,24].

The Bland-Altman analysis presented in Fig 5 showed that there was no relation between the differences and the means of the logarithmically transformed SVs obtained from the model and PhysioFlow. For HD patients, the mean difference of the log-transformed SVs was -0.41. The antilog of this difference gives a dimensionless ratio of 0.66, with the relatively wide 95% limits of agreement equal to 0.30 and 1.42. Thus, the model-based estimates of SV were, on average, 34% lower than the bioimpedance-based values from PhysioFlow, with approximately 95% of results being up to 42% higher and up to 70% lower than the reference values.

Bikia et al. used a similar model but with a more advanced arterial bifurcation tree to estimate the cardiac output (CO) and central systolic blood pressure, based on brachial systolic and diastolic pressure (brSP, brDP) and carotid-to-femoral pulse wave velocity (cf-PWV) [11]. Their approach is based on the assumption that the parameters describing arterial compliance, total peripheral resistance, and maximal blood flow from the left ventricle may be unambiguously estimated from brSP, brDP, and cf-PWV. When they adjusted their model to match brSP, brDP and cf-PWV obtained with SphygmoCor, they achieved a relatively high correlation between the model-estimated CO and the reference CO obtained using 2–D transthoracic echocardiography (r = 0.73), although they studied healthy subjects only (n = 20).

Since HD patients have a very high rate of cardiovascular morbidity and mortality [2,25], similar research in these patients is of utmost importance. Therefore, in our study, we sought to investigate whether a model-based estimation of SV is possible when based on data from HD patients, whose hemodynamics may be impaired due to both CKD and HD treatment. The main difference between our approach and that of Bikia et al. [11], is that we used the entire shape of the peripheral pressure waveform measured by SphygmoCor to inform the model. To our knowledge, no such study has been conducted to date.

The factors leading to the increased incidence of CVD in HD patients are complex and not fully understood [26]. Mathematical models may help explain these relationships. Subject-specific models can allow for an in-depth analysis of the physiological state of the patient using only non-invasive diagnostic tools. In particular, non-invasive estimation of stroke volume or left-ventricular end-systolic elastance, *E*_*max*_, can provide useful information about the condition of the heart. The scaling factor *S*_*R*_, in turn, may shed some light on the level of peripheral resistance, which is mainly influenced by the resistance of small arteries and arterioles. Tracking changes in the aforementioned parameters for a given patient could help in the early detection of CVD or in the monitoring of CVD progression, and hence it could improve treatment outcomes.

The analysis of correlations between parameters obtained from our model and those derived by SphygmoCor showed that, for both HD and control groups, there was a significant correlation between the maximal (end-systolic) elastance (*E*_*max*_) and systolic pressure (both central and peripheral) and between *E*_*max*_ and the end-systolic pressure estimated for the central pressure waveform. *E*_*max*_ is generally load-independent (just a little dependent on the arterial load) and is determined by cardiac muscle contractility and ventricular wall mass [27]. It is often approximated by the slope of the line connecting the top-left corners of the cardiac pressure-volume loops [27]. In some studies, the authors underlie that this relation is more curved than straight, which suggests that *E*_*max*_ may be dependent on the pressure and volume of the left ventricle [28,29]. This in turn may explain the correlation that we observed between the end-systolic elastance and systolic pressure. Interestingly, in HD patients we observed a moderate correlation between *E*_*max*_ and diastolic pressure (both central and peripheral) that was not present in the control group. Moreover, we observed a low positive correlation of *E*_*max*_ with weight, height or BMI in HD patients, and no such correlations in the control group.

## Limitations

Mathematical modeling of complex systems, such as the cardiovascular system, typically involves many simplifications and thus limitations. In our previous study, we have mentioned the possible limitation related to the fact that our model does not account for the presence of an arteriovenous fistula, which may influence the model-derived parameters [19]. Another limitation is related to the selection of parameters tuned in the optimization procedure, while leaving other parameters fixed. Moreover, in our model, we consider only the work of the left ventricle, with the pressure in the left atrium fixed at a constant level, which makes it impossible to take into account the Frank-Starling mechanism. Furthermore, an important limitation of our study is the possibly low reliability of some of the reference SV values obtained with PhysioFlow in HD patients. Transthoracic bioimpedance methods are cheap, non-invasive, and relatively easy to use. However, some studies challenge the accuracy of PhysioFlow, e.g., in patients with chronic anemia [23] or pediatric patients [24]. To properly validate our model, future studies should ideally use gold-standard invasive methods for SV estimation, such as the direct Fick method. Also, a more in-depth sensitivity and identifiability analysis should be performed to potentially improve the selection of parameters to estimate.

## Materials and methods

### Ethics statement

The study was approved by the Bioethical Committee at the Medical University of Lublin (Poland), and informed verbal consent has been obtained from all subjects. Our study was performed in accordance with the Declaration of Helsinki and all applicable regulations.

### Study subjects

We studied two groups: 1) the control group consisting of 14 healthy subjects, 2) the HD group, consisting of 35 anuric, prevalent hemodialysis patients, i.e. patients with end-stage renal disease, monitored during two standard HD sessions: after a long (3-day) and a short (2-day) interdialytic break, i.e. the time since the previous HD session. All HD patients had arteriovenous fistulas. None of the patients were diagnosed with CVD at the time of the study. For more detailed information on the studied HD patients, please see our previous work [19] and Table 3.

**Table 3:** Characteristics of the study subjects. Data are reported as means ± standard deviation. The data reported for hemodialysis (HD) patients were assessed after the mid-week HD hemodialysis session.

	Unit	HD patients	Controls
		N = 35	N = 14
Gender	% (male)	43	43
Age	years	61.2 ± 14.3	45.3 ± 12.0***
Height	cm	167.9 ± 9.4	171.2 ± 6.8
Weight	kg	72.2 ± 19.9	77.4 ± 13.2
Ethnic origin	% (white Caucasian)	100	100

*** p < 0.001; Mann-Whitney test

### Measurements

The pressure wave measurements were performed by one trained clinician (on the non-fistula arms in HD patients) using applanation tonometry (SphygmoCor, AtCor Medical, Australia). The results were calibrated with the systolic and diastolic blood pressure measured oscillometrically at the brachial artery (Omron M3, Omron Healthcare, Kyoto, Japan). Based on the “operator index” of the SphygmoCor device, we excluded from the analysis all low-quality recordings (according to the manufacturer’s user manual, the results are acceptable when the operator index is ≥ 80).

**Fig 8:**
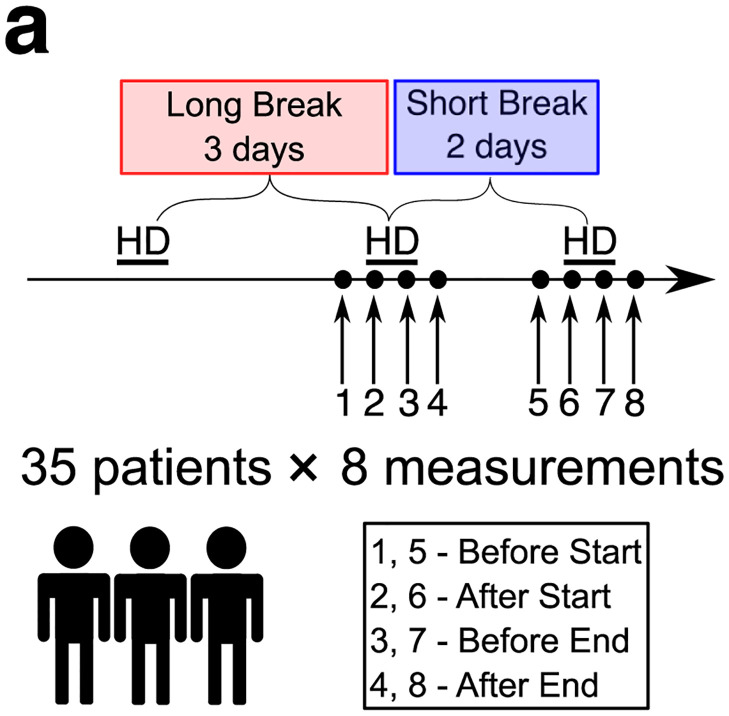
Graphical summary of the timeline of measurements in HD patients. Measurements of the pulse wave in the radial artery were performed in 35 HD patients at 4 time points during two HD sessions (after a long and a short interdialytic break). In the control group the measurements were performed at one time only.

For each subject from the control group, one to three measurements were taken, and the measurement with the best operator index was selected for further analysis. For the HD group, in each patient, 8 recordings of the pressure waveform in the radial artery were performed. The waves were recorded about 15 minutes before the start, after the start, before the end and after the end of two HD sessions, and took about 2 minutes each, see Fig 8. In 18 patients, the measurements were performed in the morning (HD starting around 7 AM), 13 patients had the measurements taken during the midday session (HD starting around 12PM), and the remaining 4 patients were measured in the evening sessions (HD starting around 6 PM). For a given patient, both studied HD sessions (i.e. after the long and the short interdialytic break) were performed at the same time of day.

The measurements of SV were performed using a non-invasive impedance cardiograph (PhysioFlow, Manatec Biomedical, France), according to the manufacturer’s protocol. The measurements were taken simultaneously with the pulse wave recordings. The quality of the obtained data was evaluated based on the “signal quality” index recorded by the device during the measurement procedure. Each bioimpedance measurement took about 2 minutes. We averaged the SVs obtained during this time, after excluding all measurements with the signal quality index not exceeding 90 on a scale from 1 to 100. According to the manufacturer, the respiratory component of the chest impedance signal is filtered out and should not affect SV estimation [30]. More detailed description of the measurement methodology can be found in [31] During all measurements the patients were lying motionless in the supine position.

The data for HD patients were divided according to the duration of the interdialytic break (a short vs long break, i.e. a two-day vs three-day break since the previous HD session), and by the moment of measurement (before the start, after the start, before the end, and after the end of the HD session), see S3 Fig.

Analyzing measurements performed at different times during moments of dialysis treatment is justified by hemodynamic changes occurring during HD. Initiating an HD session typically leads to a decrease in arterial blood pressure and workload on the heart. This is attributed to the reduction in the volume of blood in the body, as part of it is redirected to fill the extracorporeal tubing and the dialyzer (assuming that the priming fluid is discarded and not infused to the patient). Typically, all further reductions in blood volume due to removal of the excess fluid in the dialyzer, result in additional reduction in the ventricular afterload, until a relatively steady state is achieved towards the end of the session (assuming no hypotensive or hypertensive episodes). The final phase of a dialysis session involves the return of blood from the extracorporeal circuit to the body, which increases the intra-body blood volume, having the opposite effects on the heart and pulse wave compared to the pre-dialysis procedure [19].

The duration of the interdialytic break, on the other hand, may be related to the level of cardiovascular risk–the longer the break, the higher the risk, which may be due to greater changes in fluid and electrolyte balance, acidity levels, or arterial wall parameters [32]. Given the ongoing debate on the mechanisms behind increased cardiac mortality rates following a three-day interval break [32], it seems desirable to investigate how cardiovascular parameters correlate with the duration of the interdialytic break.

### The cardiovascular model

We propose a one-dimensional bifurcation arterial tree model consisting of 55 compliant arteries coupled with zero-dimensional boundary conditions representing the downstream vessels. The bifurcation tree represents the most important arteries in the human body and has been frequently used to analyze the hemodynamics of the human cardiovascular system [10,14,16,33]. We assumed that blood is an incompressible, Newtonian fluid, flowing with a parabolic velocity profile through axisymmetric elastic cylinders that taper along their length. The equations describing the blood flow and pressure were derived by integrating the incompressible longitudinal components of the Navier-Stokes equations over the vessel cross-sectional area. To close the system and ensure the uniqueness of the solution, we included the following state equation:

$$P\left(x,t\right)-P_{0}=f\left(x\right)\left(1-\frac{A_{0}\left(x,t\right)}{A\left(x,t\right)}\right)$$ (1)

which relates the blood pressure *P* (at distance *x* and at time *t*) to the cross-sectional area *A* of the vessel (*A*_0_ is the cross-sectional area of the vessel at the nominal pressure *P*_0_). In the below equation, we define the elasticity function of the arteries analogously to how it was done by Olufsen et. al. [16],

$$f\left(x\right)=\frac{4}{3}\left(k_{1}+exp\;\left(-k_{2}r_{0}\left(x\right)\right)+k_{3}\right),$$ (2)

where parameters *k*_*i*_ are global, i.e., the same for each artery and

$$r_{0}\left(x\right)=r_{in}{\left(\frac{r_{out}}{r_{in}}\right)}^{x/L},$$ (3)

describes the vessel’s tapering at the nominal pressure *P*_0_ with *r*_*in*_, *r*_*out*_ being the proximal and distal radii of the artery, and *L*–the length of the artery. At the distal ends, as an outflow boundary condition, we consider the 3-element Windkessel model, which may be expressed by the following formula describing the relation between the terminal flow *Q*_*end*_ and pressure *P*_*end*_ [34],

$$R_{1}R_{2}C_{T}\frac{dQ_{end}\left(t\right)}{dt}=R_{2}C_{T}\frac{dP_{end}\left(t\right)}{dt}+\left(P_{end}\left(t\right)-P_{T}\right)-\left(R_{1}+R_{2}\right)Q_{end}\left(t\right),$$ (4)

where, *R*_1_, *R*_2_ are the proximal and distal resistances, respectively, *C*_*T*_ is the total compliance of the terminal vascular branch, *P*_*T*_ is the reference terminal pressure. It was assumed, that *R*_1_/*R*_*T*_ = 0.2 [35], where *R*_*T*_ is the total resistance of the terminal vascular branch (*R*_1_ + *R*_2_ = *R*_*T*_,). The values of *R*_*T*_, *C*_*T*_ were taken from [36]. The 3-element Windkessel model is directly connected to the 1D model using the “ghostpoint” method, described in [9,37].

We assume that there are no blood leakages or energy losses at the vessel junctions, which are all characterized by mass conservation and pressure continuity equations:

$$Q_{end,p}=Q_{in,d_{1}}+Q_{in,d_{2}},\;\text{and}\;P_{end,p}=P_{in,d_{1}}=P_{in,d_{2}},$$ (5)

where *p* denotes the parent vessel and *d*_1_, *d*_2_ are the daughter vessels. In reality, there may be some loss of energy at the bifurcations due to the formation of vortices. However, it was shown that assuming pressure continuity is a good approximation for the considered system [15,16,38].

In our previous study, we used a phenomenological inflow boundary condition to describe the work of the heart, as previously proposed by Olufsen [36]. In the present study, to validate the model-based estimations of stroke volume (SV), we decided to use a more accurate description of the aortic inflow based on the elastance function of the left ventricle [39]. For simplicity, we decided not to modify our model of the cardiovascular system by including the arteriovenous (AV) fistula for HD patients. As shown in our previous work, the pulse wave analysis (PWA) is not significantly affected by the presence of an AV fistula, which has only a little effect on the radial-to-aortic transfer function [19].

Because we do not model the venous return to the heart, it is sufficient for the inflow boundary condition to consider only the left-ventricular function. According to the work of Suga et.al. [40] and Danielsen and Ottesen [39], the relation between the pressure in the left ventricle *P*_*lv*_ and ventricular volume *V*_*lv*_ may be described by the following equation:

$$P_{lv}=E_{lv}\left(t\right)\left(V_{lv}-V_{0}\right)$$ (6)

where *V*_0_ is the volume of the left ventricle at the zero transmural pressure and *E*_*lv*_(*t*) is a time-varying elastance function of the left ventricle, which can be approximated by the following formula

$$E_{lv}\left(t\right)=E_{min}\left(1-\phi \left(t\right)\right)+E_{max}\phi \left(t\right)$$ (7)

where

$$\phi \left(t\right)=\;\left\{\begin{array}{cc} a\;\text{sin}\left(\frac{\pi t}{t_{m}}\right)-b\;\text{sin}\left(\frac{2\pi t}{t_{m}}\right) & \;\text{for}\;0\leq t<t_{m}\\ 0 & \;\text{for}\;t_{m}\leq t<T \end{array}\right..$$ (8)

The parameters *E*_*max*_ and *E*_*min*_ are the maximal (systolic) and minimal (diastolic) values of the elastance function, *T* denotes the heart period, and *t*_*m*_–the time until the onset of constant (minimal) elastance. The parameters *a* and *b* describe the shape of *E*_*lv*_ function, see S4 Fig for an exemplary plot of the elastance function.

During the isovolumic relaxation phase, both valves in the left ventricle are closed. The pressure in the left ventricle *P*_*lv*_ decreases according to the formula (6), until it is lower than the pressure in the left atrium (*P*_*la*_, constant in our model), that is when the mitral valve opens and the ventricular filling begins. The flow between the left atrium and left ventricle *Q*_*la*_ is described as follows:

$$\frac{dQ_{la}}{dt}=\frac{1}{L_{la}}\left(P_{la}-P_{lv}\right)-\frac{R_{la}}{L_{la}}Q_{la},$$ (9)

where *L*_*la*_ is an inertia term and *R*_*la*_ denotes ventricular filling resistance caused mainly by the viscous properties of the blood. Simultaneously, the volume of the left ventricle increases, according to the formula

$$\frac{dV_{lv}}{dt}=Q_{la}.$$ (10)

Once the volume *V*_*lv*_ is greater than the maximal volume of the left ventricle, the mitral valve closes (which implies that *Q*_*la*_ returns to 0) and the isovolumic contraction begins.

During this phase, both the mitral and aortic valves are closed and the ventricle contracts, which implies that the pressure in the left ventricle increases. When *P*_*lv*_ > *P*_*a*_, where *P*_*a*_ is the root aortic pressure, the aortic valve opens, and the heart starts to eject blood into the ascending aorta. The flux between the left ventricle and aorta, *Q*_*lv*_, is given by a formula similar to (9),

$$\frac{dQ_{lv}}{dt}=\frac{1}{L_{lv}}\left(P_{lv}-P_{a}\right)-\frac{R_{lv}}{L_{lv}}Q_{lv},$$ (11)

where *P*_*a*_ is taken directly from the 1D model of the arterial tree. Concurrently, the volume *V*_*lv*_ decreases:

$$\frac{dV_{lv}}{dt}=-Q_{lv}.$$ (12)

During the aortic valve closure (which begins at time t*), some blood flows back to the left ventricle (negative *Q*_*lv*_). We allow for a certain volume of the backflow $\bar{V_{b}}$, before the complete closure of the aortic valve. When the backflow volume *V*_*b*_ given by

$$V_{b}=\int_{t^{*}}^{t}\left|Q_{lv}\right|,\;\;t>t^{*}$$ (13)

exceeds $\bar{V_{b}}$, the aortic valve closes, which implies that *Q*_*lv*_ = 0, and the cycle repeats.

The above equations and conditions fully describe the blood flow in the modelled system. The governing equations of the blood flow in the 1D domain were solved using the Lax-Wendroff scheme [41]. The inflow and outflow boundary conditions were connected to the 1D model using the “ghost point” method [9,37]. The inflow boundary condition was solved iteratively, i.e. after solving the equations of the 1D model, the elastance model of the left ventricle used the computed pressure, *P*_*a*_, from the root of the ascending aorta. Then, using the Runge-Kutta scheme, Eqs (6)–(12) were solved. At this point, we had information about the outflow from the left ventricle and the cross-sectional area at the root of the ascending aorta. In the same iterative step, using the explicit Euler method, we solve Eq (4) characterizing the outflow boundary conditions.

### Parameter estimation procedure

The bifurcation tree we use describes the vascular system for a 175 cm tall man. To personalize the vascular domain for a given subject, we multiply the lengths of all arteries, along with their proximal and distal lumen radii by the scaling factor *S* defined as the ratio of the subject height to the default height of 175 cm. Because the values of the resistances and compliances of the terminal branches depend on, among others, the size of the vessels, we also scale their default values by 1/*S*^3^ and *S*^3^ respectively, similarly as done in our previous studies [10,19]. The parameters *k*_1_, *k*_2_ and *k*_3_, which describe the stiffness of arteries were taken from the work of Olufsen [36]. The distending pressure *P*_0_ from the Eq (1) was set at the level of 97 mmHg [15]. In the elastance model of the left ventricle [23] the initial end-systolic left ventricular volume, *V*_*lv*_, was set to 120 ml and then scaled by *S*^3^. In a similar manner we scaled the volume of the left ventricle at zero pressure, *V*_*o*_ with the default value of 15 ml. The values of the heart resistances and inertances, (*R*_*lv*_, *R*_*la*_, *L*_*lv*_, *L*_*la*_) as well as the values describing the shape of the elastance function (*a*, *b*, *E*_*min*_) and the assumed volume of the backflow, $\bar{V_{b}}$, were taken from [39] or assumed. All aforementioned parameters remain constant in our model, and their values can be found in the S2 File.

Parameter estimation was carried out in two steps. In the first step, we used the meta-heuristic Particle Swarm Optimization (PSO) method [42]. The optimization started with 15 particles (a swarm), each representing a different combination of the values of the four patient-specific parameters being estimated. Then, these particles iteratively explored the search space to find a near-optimal solution (i.e., to minimize the error function). In our case, we set up 10 iterations, during which, in simple terms, the position of each particle was modified based on its own best previous position and based on the best previous position within the whole swarm, looking for the global minimum of the error function. In the second step, the best set of parameter values obtained with PSO served as the starting point for the gradient-based algorithm (GBA) [43,44], complementing and refining the previous optimization method.

The following parameters were estimated: the scaling factor of terminal resistances *S*_*R*_, the scaling factor of terminal compliances, *S*_*C*_, maximal systolic elastance, *E*_*max*_, and *t*_*m*_ denoting the time to the onset of constant elastance. The rationale behind the choice of these parameters is presented in the S2 File.

The model fitting procedure was analogous to the one used in our previous studies [10,19] but with a different error function. We defined the objective function as the sum of squares of differences between the parameters of the Fourier series expansion of the measured and model-simulated pressure waveforms. More precisely, for each measured and simulated pressure waveform in the radial artery, we approximated the first six parameters of the following sine-cosine Fourier series expansion, thus obtaining the following time-dependent signals:

$$signal\approx \frac{a_{0}}{2}+\sum_{i=1}^{6}\;\left[a_{n}\;cos\;\frac{2\pi t}{T}+b_{n}\;sin\;\frac{2\pi t}{T}\right],$$ (14)

where *T* is the heart period, and then for each case we defined the following 13-element vectors:

$$c=\left[a_{0},a_{1},\ldots ,a_{6},b_{1},\ldots ,b_{6}\right].$$ (15)

The error function was defined as follows:

$$err=\sum_{i=1}^{13}\;{\left(c_{s,i}-c_{m,i}\right)}^{2},$$ (16)

where subscripts *s* and *m* denote simulation and measurement, respectively. The optimization procedure involved minimizing that error by changing the values of the four selected model parameters. A simplified workflow of the study is presented in Fig 1. The number of analyzed harmonics has been chosen arbitrarily and represents a compromise between the accuracy of the pulse wave representation and the complexity of the objective function; S5 Fig presents some examples of Fourier-transformed pressure waveforms for healthy subjects and HD patients.

### Statistical methods

Statistical dependence between *in vivo* data and the model-estimated parameters was measured using Pearson correlation coefficient (r). Statistical differences between the paired data were investigated using the Wilcoxon signed-rank test. Statistical significance was set at the level of p = 0.05. To verify normal distribution of differences between the measured and estimated SV, the Shapiro-Wilk test was performed. The accuracy of model-simulated pressure waveforms (with respect to the recorded waveforms) was assessed by mean absolute percentage error (MAPE). The regression lines were plotted with 95% confidence intervals.

## Conclusions

We investigated the feasibility of using a patient-calibrated 0-1D model of the systemic circulation to estimate SV. The preliminary validation of the model in a group of 35 HD patients and a control group of 14 healthy subjects showed that the model was able to reproduce the pressure waveforms recorded non-invasively in the radial artery with satisfactory accuracy in most cases and that the model-based estimates of SV are correlated with the reference bioimpedance-based SV estimates. However, the model seems to underestimate SV in both HD patients and healthy subjects. This may be at least partly an apparent result, given the possible inaccuracy of the reference bioimpedance measurements. If more accurate reference measurements confirm that the model does, indeed, underestimate SV, this could be due to: 1) limitations of our model, 2) too few fitted parameters of the model, or 3) a combination of the above factors. To fully and properly validate the model, a larger study should be conducted with a more accurate, gold-standard reference method for estimating SV, possibly with a larger number of model parameters to be fitted, selected after a more in-depth sensitivity and identifiability analysis.

## Supporting information

S1 File Supporting materials. The file presents simulated vs measured pressure waveforms in the radial artery.
(PDF)

S2 File Supporting materials. The file presents the rationale behind the choice of model parameters to be fitted, a sensitivity analysis, as well as justification that the selected parameters uniquely determine the pulse wave.
(PDF)

S1 Fig Exemplary outputs from the model at various locations of the arterial tree. The presented model outputs (i.e. blood pressure and flow rate waveforms) were obtained using the baseline values of all model parameters (see S1 supplementary materials), for a 175 cm man with a heart rate of 75 bpm.
(PNG)

S2 Fig **(a) Pearson correlation coefficients between the model-estimated (computed) SV and the SV measured using bioimpedance cardiography for HD patients (additionally divided into groups of measurements depending on the duration of the interdialytic break before the studied HD session and the moment of measurement during the HD session). (b) Scatter plots of model-estimated (computed) and bioimpedance-based SV values for HD patients corresponding to different moments of measurement**. Correlations were computed after removing the outlier from the Before End group.
(PNG)

S3 Fig Sankey diagram of the study dataset collected in HD patients. 136 cases were excluded from the analysis due to missing or clearly erroneous data or due to low quality of the recorded applanation tonometry or bioimpedance signals in accordance with the manufacturer’s instructions (SphygmoCor “Operator index” < 80 or PhysioFlow “Signal Quality” < 90). The remaining cases were divided according to the length of the interdialytic break before the studied HD session (a short, 2-day break vs a long, 3-day break) and according to the time of measurement during the HD session (before start, after start, before end and after end of the HD).
(PNG)

S4 Fig Left-ventricular elastance function according to Eqs (7) and (8). *E*_*max*_–maximal systolic elastance, *E*_*min*_–minimal (diastolic) elastance, *T*–heart period, and *t*_*m*_–time to the onset of constant (minimal) elastance.
(PNG)

S5 Fig Exemplary pressure waveforms recorded in the radial artery by SphygmoCor along with their transformations into Fourier series with 6 harmonics. Results are presented for two healthy subjects and two HD patients and normalized against time, *T*–heart period.
(PNG)
