# Supplementary material for: Non-invasive assessment of stroke volume and cardiovascular parameters based on peripheral pressure waveform
Source: PLoS Comput Biol. 2024 Apr 18;20(4):e1012013. doi: 10.1371/journal.pcbi.1012013 (PMC11060565; doi:10.1371/journal.pcbi.1012013)
Supplement: S1 File — The file presents simulated vs measured pressure waveforms in the radial artery. (PDF) [file pcbi.1012013.s001.pdf]

Supplementary materials for the article

"Non-invasive assessment of stroke volume and cardiovascular parameters based on peripheral pressure waveform"

by

Kamil Wolos, Leszek Pstras, Malgorzata Debowska, Wojciech Dabrowski, Dorota Siwicka-Gieroba, Jan Poleszczuk

Choice of patient-specific parameters to be estimated and their identifiability

Compared to our previous work (1,2), in the present study we decided to limit the number of subject-specific parameters to be estimated to just four (described below), for two reasons. First, we wanted to ensure their identifiability, and second, we wanted to verify whether even such a low number of fitted parameters would allow for an assessment of patient-specific stroke volume and other parameters. One needs to remember, that the lower the number of estimated parameters, the faster the computations required to calibrate the model with data – an aspect important when thinking about future method implementation. The choice of parameters to be estimated was not a trivial matter. On the one hand, these parameters should enable a reasonably accurate fitting of the model to the recorded pressure waveforms in the radial artery. On the other hand, they should influence stroke volume (SV), the estimation of which was especially important in our study, given that this was the model output that was subject to validation. Therefore, we decided to estimate two parameters related to the time-varying left-ventricular elastance, E_{max}, and the time to the onset of constant elastance,t_m) and two parameters related to the peripheral arterial resistance and compliance, i.e. the scaling factors for the resistances and compliances of the terminal branches of the arterial tree (S_R and S_C).

The choice of the ventricular elastance-related parameters (E_{max} and t_m) was based on the observation that the left-ventricular elastance functions normalized by the time of the peak and its amplitude are similar in healthy and diseased hearts (3). The only differences between an intact and diseased heart should be in the peak value of the left-ventricular elastance (E_{max}) and the time of that peak (3). The latter is not used explicitly in our model, which is why we decided to estimate a slightly different time-related parameter used in our definition of the time-varying elastance function (see eq. (7) and

supplementary Figure S6 for more details), i.e. the time to the onset of constant (minimal) elastance, t_m, which we expect should be correlated with the time of the peak elastance. The remaining parameters of our elastance function, i.e. the parameter E_{min}, which describes the minimal left-ventricular diastolic elastance and parameters a and b, which are mainly responsible for the slope of $E_{lv}(t)$ have been assumed fixed, given the aforementioned regularity in the normalized shape of $E_{lv}(t)$.

The other two parameters chosen to be estimated are the scaling factors for the terminal resistances and compliances. The former is strictly related with the systemic resistance, which results mainly from the resistance of small arteries and arterioles (4) and has a crucial impact on arterial blood pressure and therefore on the heart function, given that the heart works against the pressure at the entry to the ascending aorta. The scaling factor for the peripheral (terminal) compliances, was chosen to be estimated given that it should affect the diastolic part of the peripheral pressure waveform and therefore should enable better overall fits to patient data.

In the future, the choice of the parameters for optimization should be addressed in a more systematic manner by analyzing various combinations of parameters used for model fitting and assessing their joint performance both in terms of fitting of the model to the recorded pressure waveforms in the radial artery as well as estimation of stroke volume using such a data-fitted model (while keeping the model identifiable). However, in order for such a systematic analysis to be robust, one should consider collecting more data, including additional measurement endpoints, preferably using gold-standard methods.

Parameter identifiability

To check if the set of the four chosen parameters can identify a pulse waveform in the radial artery, we performed an identifiability analysis based on the work of Brun et. al. (5). As the output from the model, we considered the Fourier expansion parameters of the computed pressure waveform, see equations (14) and (15) for more details.

We used the finite difference approximation to compute the local sensitivity matrix $V = \{v_{i,j}\}$ where each column v_j represents changes in model outputs caused by a small change in the value of parameter

$\theta_j \in \{E_{max},\ t_m,\ S_R,\ S_C\}$ with respect to its baseline level, θ_0, as presented in Supplementary Table 1. For each studied parameter, we increased its baseline value by 5%, leaving all other parameters unchanged (the elements of the vector v_j correspond to the elements of the vector c defined in equation (15), and j denotes the parameter being changed). Then, the scaled sensitivity matrix $S = \{s_{i,j}\}$ was computed as follows to obtain dimension-free sensitivity information:

$$s_{i,j} = \frac{v_{i,j}\Delta\theta_j}{MV_i}, \quad (17)$$

where, according to Brun et al. (5), $\Delta\theta_j$ should be an *a priori* measure of the reasonable range of θ_j, and the scale factor, MV_i, should have the same scale as the corresponding output $v_{i,j}$. Here we set $\Delta\theta_j = \theta_0$, (see Supplementary Table 1), assuming that for each parameter the expected range of its values is ±50%. For MV_i, we first calculated the Fourier expansion coefficients of all recorded pressure waveforms, as in equation (14), to obtain 13-element vectors as given by equation (15), and then we calculated the mean value (MV) of each element in those vectors and assigned it to the corresponding parameter MV_i.

SUPPLEMENTARY TABLE 1
PARAMETERS $\Delta\theta_j$ USED IN IDENTIFIABILITY ANALYSIS

Parameter	Unit	$\theta_0 = \Delta\theta_j$
E_{max}	mmHg/ml	2.49[a]
t_m	s	0.41
S_R	-	1
S_C	-	1

[a] based on the work of Danielsen (6)

To assess the identifiability of a given set of parameters, we must consider the joint influence of these parameters on the model output. This can be done by checking the degree of near-linear dependence between the columns of the scaled sensitivity matrix. To that aim we calculated a collinearity index γ defined as in (5)

$$\gamma = \frac{1}{min_{||\beta||=1}||\tilde{S}\,\beta||} = \frac{1}{\sqrt{\lambda_k}}, \quad (18)$$

where $\tilde{S}$ is the normalized sensitivity matrix S, β is a 13-element vector, and λ_k is the smallest eigenvalue of $\tilde{S}^T\tilde{S}$. According to Brun et al., the parameters may be considered identifiable, if $\gamma < 20$. In our case, $\gamma = 2.1$. On this basis, we concluded that our model is identifiable and that the obtained solutions are unique.

Sensitivity analysis

We have also investigated changes in the simulated flow rate waveform in the ascending aorta and the pulse waveform in the radial artery following changes in parameter values by $\pm 50\%$ for E_{max}, S_C and S_R, and $\pm 10\%$ for t_m, similarly as done in (7). For t_m we studied smaller changes, because 50% of the baseline value of t_m would fall below our accepted lower bound on this parameter which was set to $0.45 \cdot T$, where T is the heartbeat period. The results of this sensitivity analysis are presented in Fig. S8.

The most prominent changes in the flow rate waveforms and pulse waveforms are visible for changes in parameters E_{max} and S_R, particularly with regard to the vertical shifts of the pulse waveform, see Fig. S6, b and c. Increasing E_{max} results in an increase of the aortic inflow, whereas the aortic inflow decreases when S_R increases. Changes in E_{max} lead to changes in the maximal and total aortic inflow, whereas changes in S_R affect mainly the timing of the blood flow from the heart. Slightly less visible is the impact of t_m variations, which mainly influence the shape of the pulse waveform in the systolic phase, especially with regard to the downslope of the pressure wave after reaching the peak systolic pressure, see Fig. S6. The parameter S_C affects the diastolic part of the pulse waveform, although its impact is relatively small, see Fig. S6d. It allows for somewhat better fitting of the recorded pulse waveforms. However, it does not cause any noticeable changes in the flow rate in the ascending aorta.

The above analysis revealed, therefore, that each of the four chosen parameters influences the shape of the pressure waveform in the radial artery and that all of them except Sc also affect stroke volume (calculated from the flow rate waveform in the ascending aorta).

Fixed parameters in the cardiovascular model

Supplementary Table 2 presents the parameters that are fixed in the model. The values of these parameters come mainly from the work of Olufsen (8) and Danielsen (6).

Supplementary Table 2
Fixed parameters of the model

Parameter	Unit	Value	Reference
E_{min}	$mmHg/ml$	0.049	(6)
a	-	0.9	(6)

b	-	0.25	(6)
k_1	$g/(s^2 \cdot cm)$	$2 \cdot 10^7$	(9)
k_2	cm^{-1}	-22.53	(9)
k_3	$g/(s^2 \cdot cm)$	$8.65 \cdot 10^5$	(9)
P_o	$mmHg$	97	(10)
R_{la}	$mmHg \cdot s/ml$	0.000089	(6)
L_{la}	$mmHg \cdot s^2/ml$	0.00005	(6)
p_{la}	$mmHg$	7.5	assumed
R_{lv}	$mmHg \cdot s/ml$	0.08	assumed
L_{lv}	$mmHg \cdot s^2/ml$	0.000416	(6)
$\overline{V_b}$	ml	2	(6)
V_o	ml	$15 \cdot S^3$	assumed

S is the ratio of the subject's height to the height of the reference man (175 cm)

REFERENCES

1. Poleszczuk J, Debowska M, Dabrowski W, Wojcik-Zaluska A, Zaluska W, Waniewski J. Subject-specific pulse wave propagation modeling: Towards enhancement of cardiovascular assessment methods. PLoS One. 2018;13(1):1–17.

2. Poleszczuk J, Debowska M, Dabrowski W, Wojcik-Zaluska A, Zaluska W, Waniewski J. Patient-specific pulse wave propagation model identifies cardiovascular risk characteristics in hemodialysis patients. PLoS Comput Biol. 2018;14(9):1–15.

3. Senzaki H, Chen CH, Kass DA. Single-Beat Estimation of End-Systolic Pressure-Volume Relation in Humans. Circulation [Internet]. 1996 Nov 15 [cited 2022 May 6];94(10):2497–506. Available from: https://www.ahajournals.org/doi/abs/10.1161/01.CIR.94.10.2497

4. Lax P, Wendroff B. Systems of conservation laws. Commun Pure Appl Math [Internet]. 1960 May 1 [cited 2024 Feb 15];13(2):217–37. Available from: https://onlinelibrary.wiley.com/doi/full/10.1002/cpa.3160130205

5. Brun R, Reichert P, Künsch HR. Practical identifiability analysis of large environmental simulation models. Water Resour Res [Internet]. 2001 Apr 1 [cited 2022 Oct 25];37(4):1015–30. Available from: https://onlinelibrary.wiley.com/doi/full/10.1029/2000WR900350

6. Danielsen M, Ottesen JT. 6. A Cardiovascular Model. Mathematical Modeling and Computation [Internet]. 2004 Jan [cited 2022 May 4];137–55. Available from: https://epubs.siam.org/doi/abs/10.1137/1.9780898718287.ch6

7. Zhang X, Liu J, Cheng Z, Wu B, Xie J, Zhang L, et al. Personalized 0D-1D multiscale hemodynamic modeling and wave dynamics analysis of cerebral circulation for an elderly patient with dementia. Int J Numer Method Biomed Eng. 2021;

8. Olufsen MS, Peskin CS, Kim WY, Pedersen EM, Nadim A, Larsen J. Numerical simulation and experimental validation of blood flow in arteries with structured-tree outflow conditions. Ann Biomed Eng. 2000;28(11):1281–99.

9. Anemette Sofie Olufsen. Roskilde University. 1998 [cited 2022 May 4]. Modeling the arterial system with reference to an anesthesia simulator. Ph.D. Thesis. Available from: https://forskning.ruc.dk/en/publications/modeling-the-arterial-system-with-reference-to-an-anesthesia-simu

10. Stergiopulos N, Young DF, Rogge TR. Computer Simulation of Arterial Flow With Applications To Arterial and Aortic Stenoses. J Biomech. 1992;25(12):1477–88.

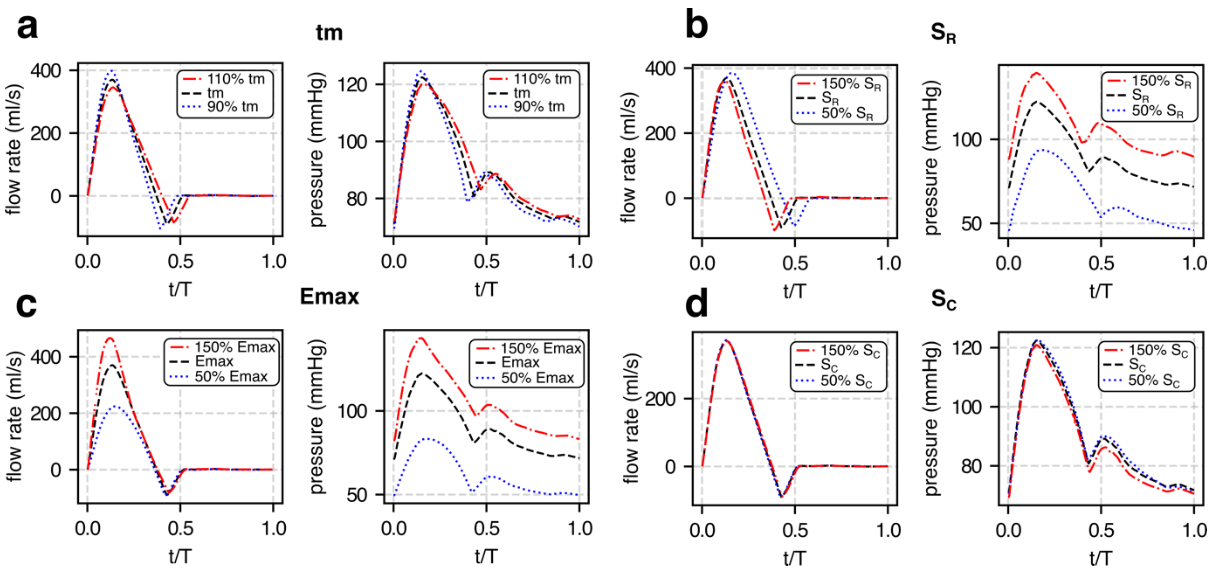

S6 Fig. Sensitivity of the simulated flow rate waveform from the ascending aorta (left subpanels) and pulse waveform in the radial artery (right subpanels) to relatively large changes in the adjustable model parameters. a) tm - time to the onset of constant elastance of the left ventricle; b) Sr – scaling factor for the resistance of the terminal branches of the arterial tree; c) Emax - maximal elastance of the left ventricle; d) Sc - scaling factor for the compliances of the terminal branches of the arterial tree.
