# Supplementary material for: Non-invasive assessment of stroke volume and cardiovascular parameters based on peripheral pressure waveform
Source: PLoS Comput Biol. 2024 Apr 18;20(4):e1012013. doi: 10.1371/journal.pcbi.1012013 (PMC11060565; doi:10.1371/journal.pcbi.1012013)

## Supplementary material 2

## Simulated vs measured pressure waveforms in the radial artery

Below we show the simulated vs measured (recorded) pressure waveforms in the radial artery (following fitting of the model to each measured waveform) for all cases analyzed in the present study, i.e. 144 measurements in 35 HD patients and 14 measurements in 14 healthy subjects (control group).

### Legend

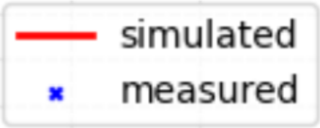


For the HD patients, the subpanels are titled using the following format:

**[ID, interdialytic break, time of measurement, error]**

where:
**ID** denotes patient ID

**interdialytic break refers to the length of break since the last HD session:**
- **SB** – short interdialytic break (2 days)
- **LB** – long interdialytic break (3 days)

**time of measurement refers to the exact moment of measurement during the HD session:**
- **BS** – measurement taken 15 min before the start of HD session
- **AS** – measurement taken 15 min after the start of HD session
- **BE** – measurement taken 15 min before the end of HD session
- **AE** – measurement taken 15 min after the end of HD session

**error** represents the fitting error, i.e. the mean absolute percentage error (MAPE) between the simulated and measured pressure waveforms (for all available data points)

**Example:**
The title "**9, SB, AS, err=2.2%**" describes the case of a patient with ID=9, with the pressure waveform measured during hemodialysis performed after a short interdialytic break and 15 min after the start of the hemodialysis session, where the MAPE between the simulated and measured pressure waveforms was 2.2%.

For the control group, the subpanels are titled using the analogous but shorter format:
**[ID, error]**

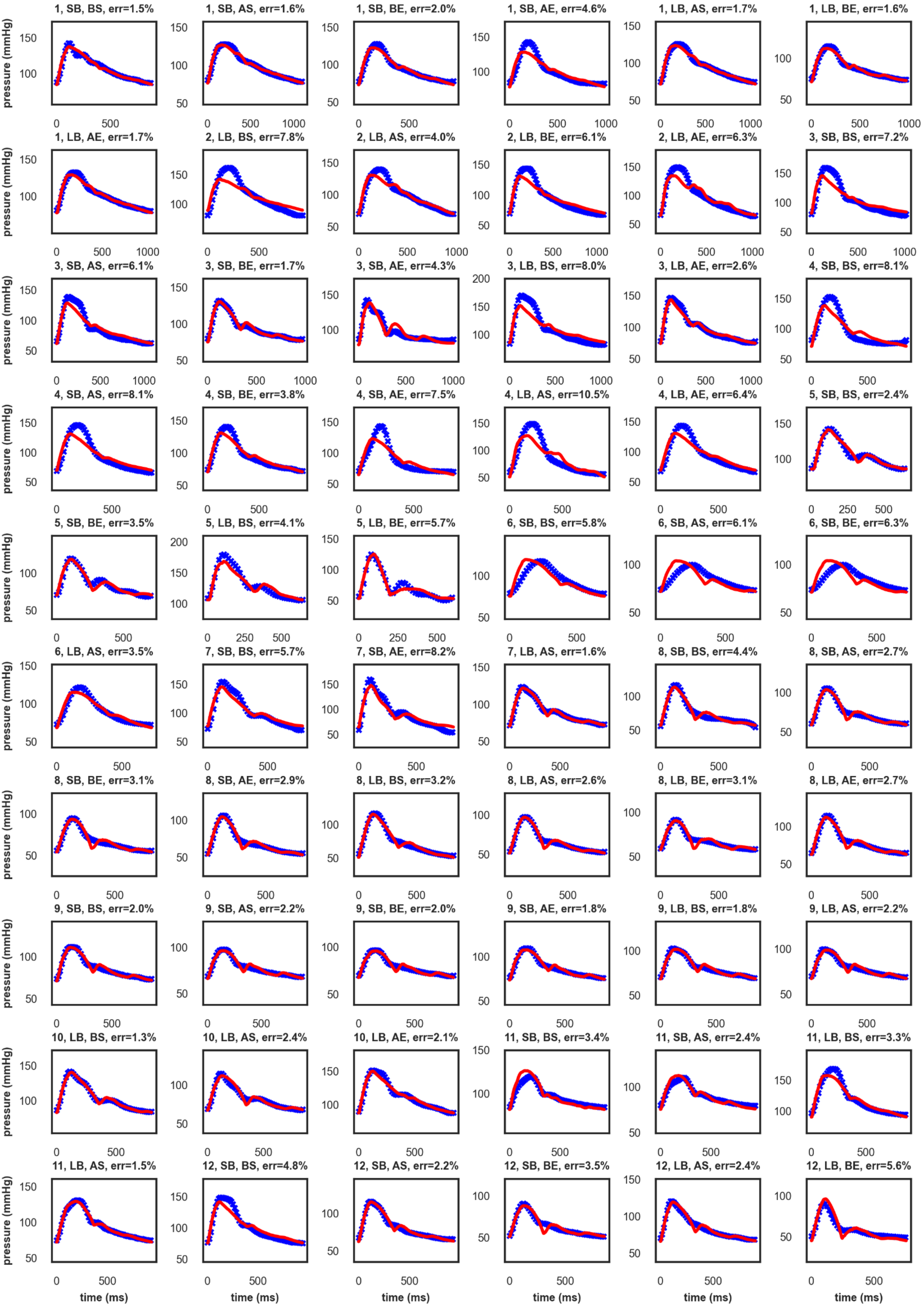
1, SB, BS, err=1.5%
1, SB, AS, err=1.6%
1, SB, BE, err=2.0%
1, SB, AE, err=4.6%
1, LB, AS, err=1.7%
1, LB, BE, err=1.6%
1, LB, AE, err=1.7%
2, LB, BS, err=7.8%
2, LB, AS, err=4.0%
2, LB, BE, err=6.1%
2, LB, AE, err=6.3%
3, SB, BS, err=7.2%
3, SB, AS, err=6.1%
3, SB, BE, err=1.7%
3, SB, AE, err=4.3%
3, LB, BS, err=8.0%
3, LB, AE, err=2.6%
4, SB, BS, err=8.1%
4, SB, AS, err=8.1%
4, SB, BE, err=3.8%
4, SB, AE, err=7.5%
4, LB, AS, err=10.5%
4, LB, AE, err=6.4%
5, SB, BS, err=2.4%
5, SB, BE, err=3.5%
5, LB, BS, err=4.1%
5, LB, BE, err=5.7%
6, SB, BS, err=5.8%
6, SB, AS, err=6.1%
6, SB, BE, err=6.3%
6, LB, AS, err=3.5%
7, SB, BS, err=5.7%
7, SB, AE, err=8.2%
7, LB, AS, err=1.6%
8, SB, BS, err=4.4%
8, SB, AS, err=2.7%
8, SB, BE, err=3.1%
8, SB, AE, err=2.9%
8, LB, BS, err=3.2%
8, LB, AS, err=2.6%
8, LB, BE, err=3.1%
8, LB, AE, err=2.7%
9, SB, BS, err=2.0%
9, SB, AS, err=2.2%
9, SB, BE, err=2.0%
9, SB, AE, err=1.8%
9, LB, BS, err=1.8%
9, LB, AS, err=2.2%
10, LB, BS, err=1.3%
10, LB, AS, err=2.4%
10, LB, AE, err=2.1%
11, SB, BS, err=3.4%
11, SB, AS, err=2.4%
11, LB, BS, err=3.3%
11, LB, AS, err=1.5%
12, SB, BS, err=4.8%
12, SB, AS, err=2.2%
12, SB, BE, err=3.5%
12, LB, AS, err=2.4%
12, LB, BE, err=5.6%
pressure (mmHg)
time (ms)

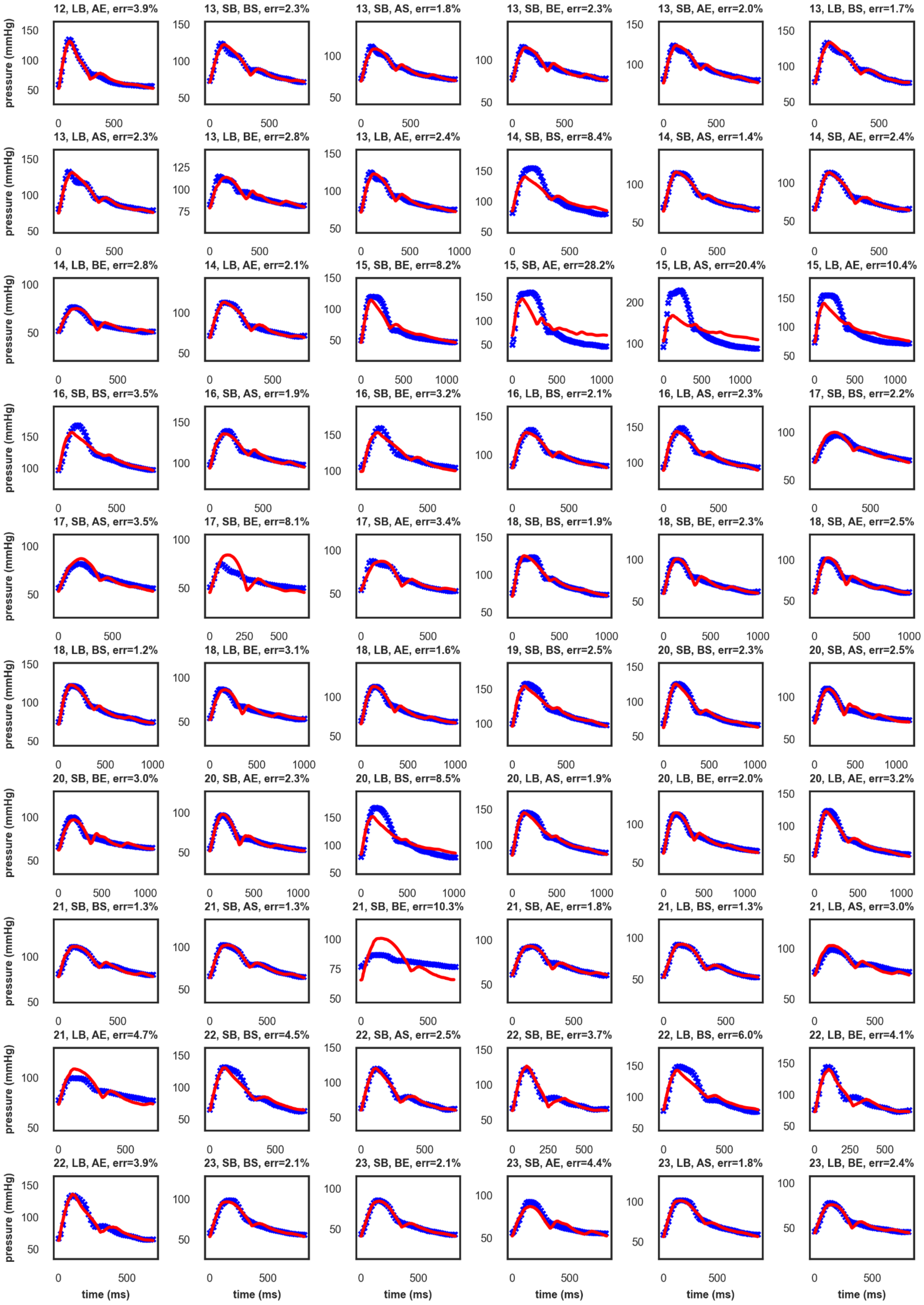
12, LB, AE, err=3.9%
13, SB, BS, err=2.3%
13, SB, AS, err=1.8%
13, SB, BE, err=2.3%
13, SB, AE, err=2.0%
13, LB, BS, err=1.7%
13, LB, AS, err=2.3%
13, LB, BE, err=2.8%
13, LB, AE, err=2.4%
14, SB, BS, err=8.4%
14, SB, AS, err=1.4%
14, SB, AE, err=2.4%
14, LB, BE, err=2.8%
14, LB, AE, err=2.1%
15, SB, BE, err=8.2%
15, SB, AE, err=28.2%
15, LB, AS, err=20.4%
15, LB, AE, err=10.4%
16, SB, BS, err=3.5%
16, SB, AS, err=1.9%
16, SB, BE, err=3.2%
16, LB, BS, err=2.1%
16, LB, AS, err=2.3%
17, SB, BS, err=2.2%
17, SB, AS, err=3.5%
17, SB, BE, err=8.1%
17, SB, AE, err=3.4%
18, SB, BS, err=1.9%
18, SB, BE, err=2.3%
18, SB, AE, err=2.5%
18, LB, BS, err=1.2%
18, LB, BE, err=3.1%
18, LB, AE, err=1.6%
19, SB, BS, err=2.5%
20, SB, BS, err=2.3%
20, SB, AS, err=2.5%
20, SB, BE, err=3.0%
20, SB, AE, err=2.3%
20, LB, BS, err=8.5%
20, LB, AS, err=1.9%
20, LB, BE, err=2.0%
20, LB, AE, err=3.2%
21, SB, BS, err=1.3%
21, SB, AS, err=1.3%
21, SB, BE, err=10.3%
21, SB, AE, err=1.8%
21, LB, BS, err=1.3%
21, LB, AS, err=3.0%
21, LB, AE, err=4.7%
22, SB, BS, err=4.5%
22, SB, AS, err=2.5%
22, SB, BE, err=3.7%
22, LB, BS, err=6.0%
22, LB, BE, err=4.1%
22, LB, AE, err=3.9%
23, SB, BS, err=2.1%
23, SB, BE, err=2.1%
23, SB, AE, err=4.4%
23, LB, AS, err=1.8%
23, LB, BE, err=2.4%
pressure (mmHg)
time (ms)

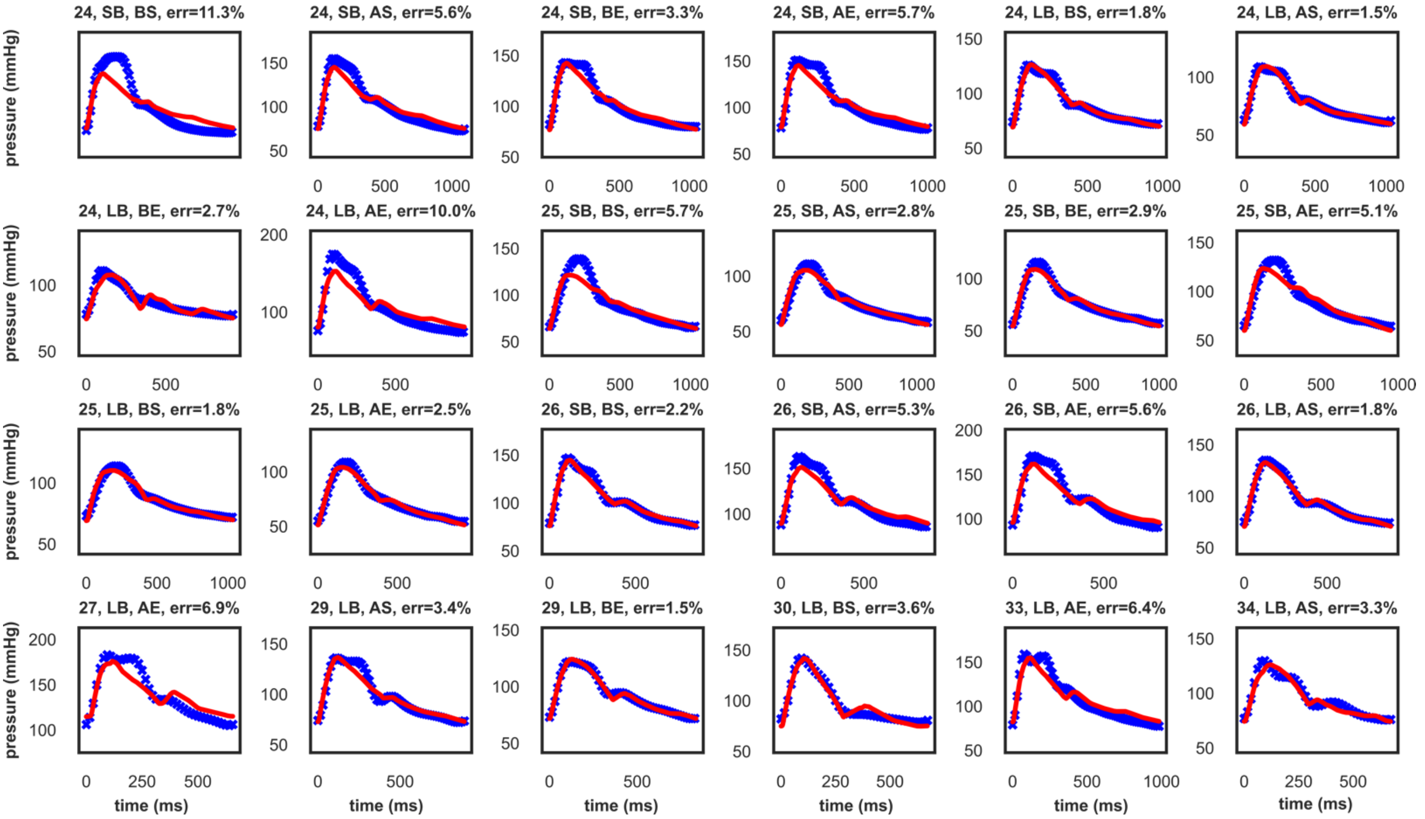


**Control group:**

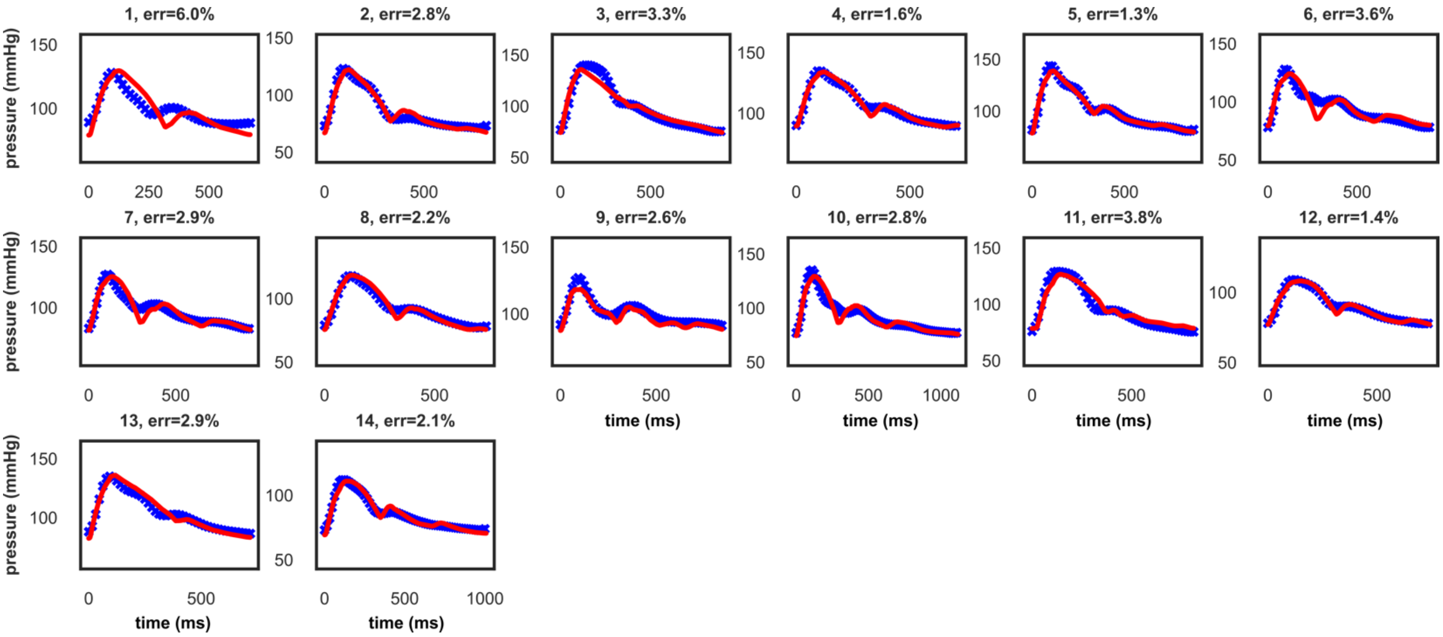

Supplement: S2 File — The file presents the rationale behind the choice of model parameters to be fitted, a sensitivity analysis, as well as justification that the selected parameters uniquely determine the pulse wave. (PDF) [file pcbi.1012013.s002.pdf]
