# Supplementary figures and images for: Non-invasive assessment of stroke volume and cardiovascular parameters based on peripheral pressure waveform
Source: PLoS Comput Biol. 2024 Apr 18;20(4):e1012013. doi: 10.1371/journal.pcbi.1012013 (PMC11060565; doi:10.1371/journal.pcbi.1012013)

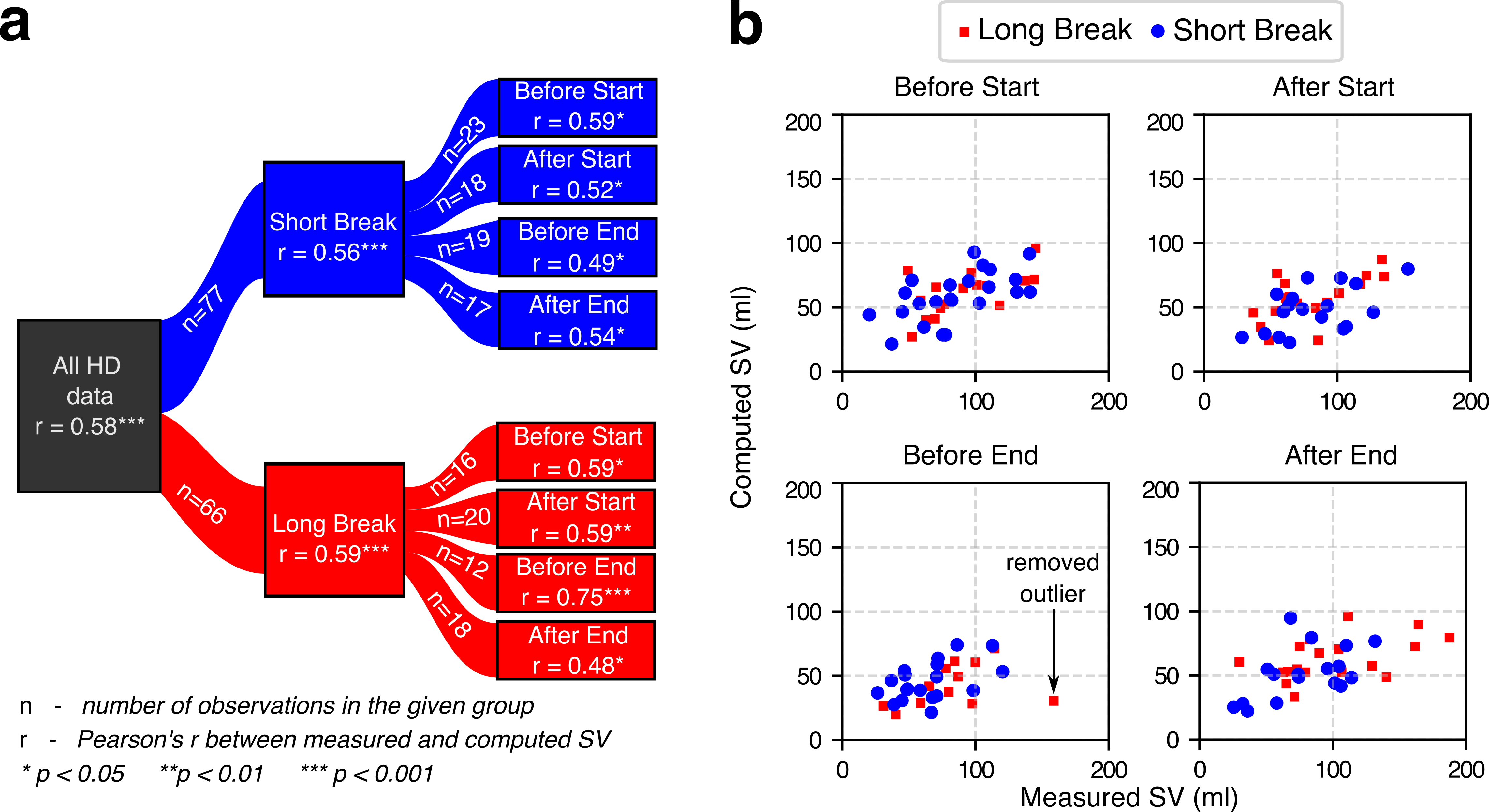

Supplement: S2 Fig — (a) Pearson correlation coefficients between the model-estimated (computed) SV and the SV measured using bioimpedance cardiography for HD patients (additionally divided into groups of measurements depending on the duration of the interdialytic break before the studied HD session and the moment of measurement during the HD session). (b) Scatter plots of model-estimated (computed) and bioimpedance-based SV values for HD patients corresponding to different moments of measurement. Correlations were computed after removing the outlier from the Before End group. (PNG) [file pcbi.1012013.s004.png]

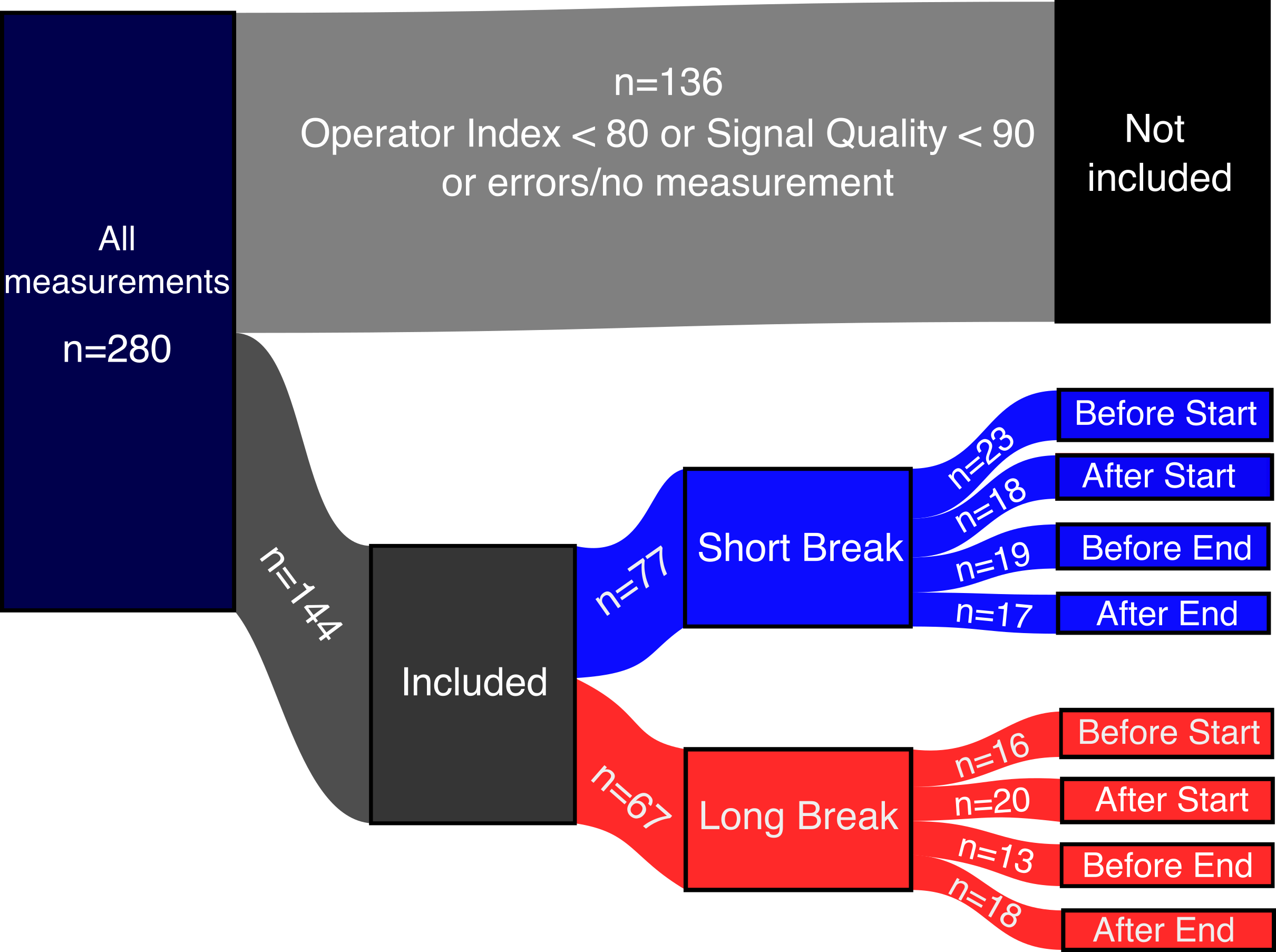

Supplement: S3 Fig — 136 cases were excluded from the analysis due to missing or clearly erroneous data or due to low quality of the recorded applanation tonometry or bioimpedance signals in accordance with the manufacturer’s instructions (SphygmoCor “Operator index” < 80 or PhysioFlow “Signal Quality” < 90). The remaining cases were divided according to the length of the interdialytic break before the studied HD session (a short, 2-day break vs a long, 3-day break) and according to the time of measurement during the HD session (before start, after start, before end and after end of the HD). (PNG) [file pcbi.1012013.s005.png]

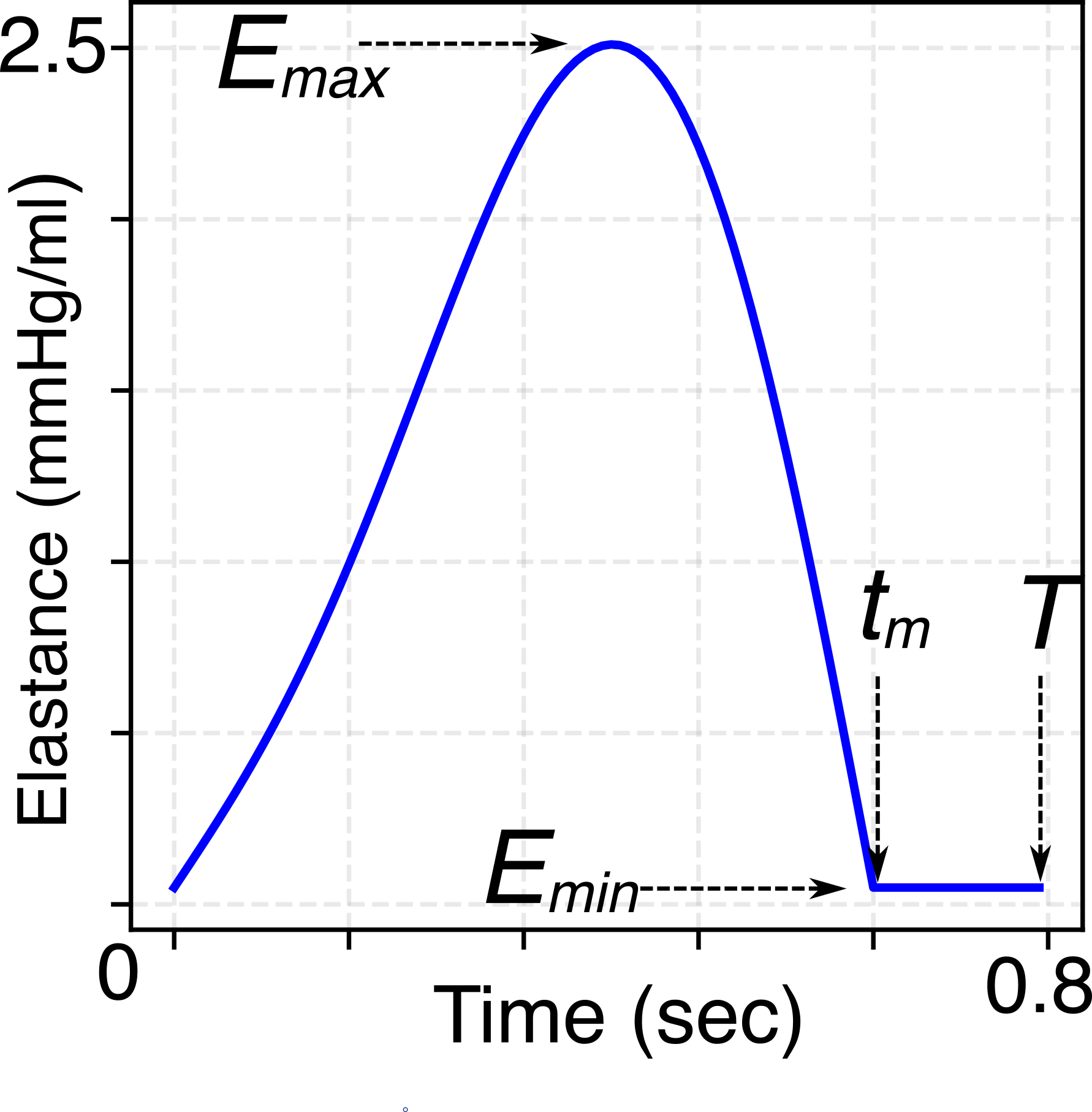

Supplement: S4 Fig — Emax–maximal systolic elastance, Emin–minimal (diastolic) elastance, T–heart period, and tm–time to the onset of constant (minimal) elastance. (PNG) [file pcbi.1012013.s006.png]

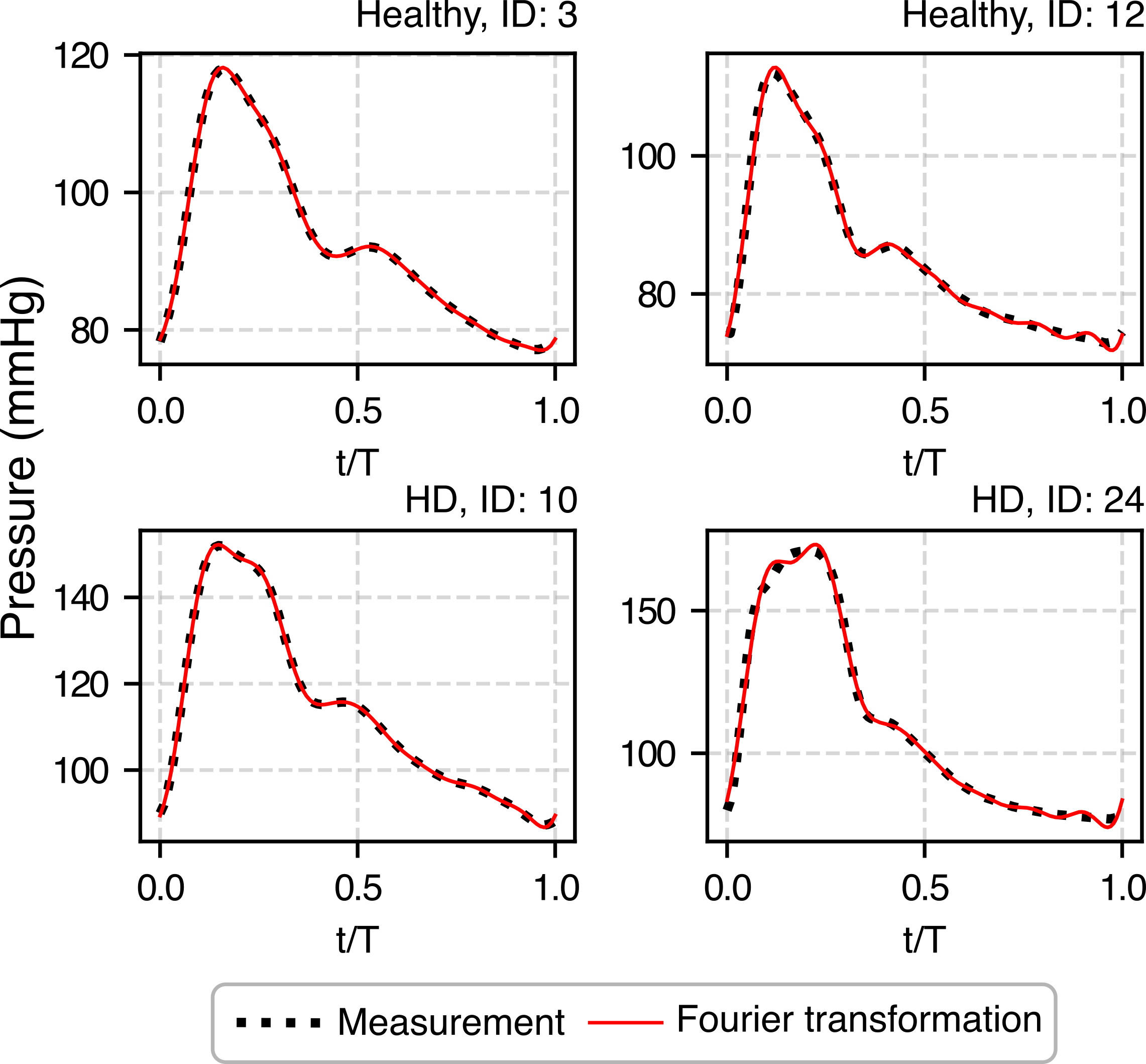

Supplement: S5 Fig — Results are presented for two healthy subjects and two HD patients and normalized against time, T–heart period. (PNG) [file pcbi.1012013.s007.png]
